# *TERT* p Mutation and its Prognostic Value in Glioma Patients Under the 2021 WHO Classification: A Real‐World Study

**DOI:** 10.1002/cam4.70533

**Published:** 2025-01-13

**Authors:** Hao Xing, Delin Liu, Junlin Li, Yulu Ge, Xiaopeng Guo, Wenlin Chen, Dachun Zhao, Yixin Shi, Yilin Li, Yaning Wang, Yuekun Wang, Yu Xia, Jiaming Wu, Tingyu Liang, Hai Wang, Qianshu Liu, Shanmu Jin, Tian Qu, Siying Guo, Huanzhang Li, Tianrui Yang, Kun Zhang, Yu Wang, Wenbin Ma

**Affiliations:** ^1^ Department of Neurosurgery, Center for Malignant Brain Tumors, National Glioma MDT Alliance Peking Union Medical College Hospital, Chinese Academy of Medical Sciences and Peking Union Medical College Beijing China; ^2^ Chinese Academy of Medical Sciences and Peking Union Medical College Beijing China; ^3^ China Anti‐Cancer Association Specialty Committee of Glioma Beijing China; ^4^ Department of Pathology Peking Union Medical College Hospital, Chinese Academy of Medical Sciences and Peking Union Medical College Beijing China

**Keywords:** adult‐type diffuse glioma, glioma classification, molecular subgroups, prognosis, *TERT* promoter mutation

## Abstract

**Background:**

The 2021 WHO Classification of Central Nervous System Tumors introduces more molecular markers for glioma reclassification, including *TERT* promoter (*TERT*p) mutation as a key feature in glioblastoma diagnosis.

**Aims:**

Given the changes in the entities included in each subtype under the new classification, this research investigated the distribution, prognostic value, and correlations with other molecular alterations of *TERT*p mutation in different subgroups under this latest classification.

**Methods:**

All glioma patients admitted to Peking Union Medical College Hospital for surgical resection or biopsy from 2011 to 2022 were included. Samples were analyzed for *TERT*p mutation and 59 other gene alterations and chromosome copy number variations.

**Results:**

A total of 207 patients were included. The occurrence of *TERT*p mutations varied with percentages of 4.55%, 100%, and 77.92% in astrocytoma, oligodendroglioma, and glioblastoma, respectively. 65% of all adult‐type glioma patients and 42.6% of IDH‐wildtype histology grade 2 or 3 patients were *TERT*p‐mutant. Survival analysis showed that *TERT*p mutation was a predictor of better prognosis in IDH‐mutant grade 2 gliomas (median OS (mOS): not reached (NA) (95% CI: NA–NA) vs. 75.9 (95% CI: 55.4–NA) months, HR = 0.077 (95% CI: 0.01–0.64), *p* = 0.003), while poor OS was associated with all Grade 4 gliomas (mOS: 17.5 (95% CI: 12.6–24.2) vs. 40.5 (95% CI: 24.4–83.8) months, HR = 2.014 (95% CI: 1.17–3.47), *p* = 0.01) and all IDH‐wildtype histology grade 2 or 3 gliomas (median OS: 12.6 (95% CI: 11–24.2) vs. 83.8 (95% CI: 35.2–NA) months, HR = 3.768 (95% CI: 1.83–7.78), *p* < 0.001). Moreover, *TERT*p mutation tended to co‐occur with *EGFR, KRAS,* and *MET* in glioblastoma. In the IDH‐mutant subgroup, it tended to co‐occur with *CIC* and *FUBP1* alterations, while being mutually exclusive with *ATRX* and *TP53* alterations. These correlations may further refine prognostic predictions.

## Graphical abstract

Given the big changes in the entities included in each subtype under the 2021 CNS tumor classification, the effect of *TERT*p mutation on prognosis and the relationship between *TERT*p mutation and other molecular alterations in different subgroups should be reevaluated. In this research, we investigated the distribution, prognostic value, and molecular correlation of *TERT*p mutation under this latest classification. Survival analysis showed that *TERT*p mutation was a predictor of better prognosis in IDH‐mutant grade 2 gliomas, while poor OS was associated with all Grade 4 gliomas and all IDH‐wildtype histology grade 2 or 3 gliomas. The correlation between *TERT*p mutation and other molecular alterations was multiform in different subgroups, which needs to be investigated further.

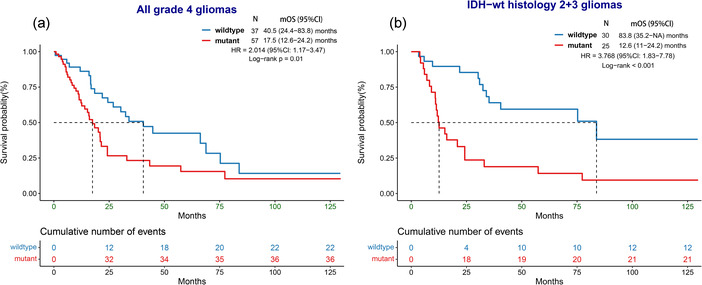

**Abbreviations**
CNS: central nervous system
GBM: glioblastoma
IDH: isocitrate dehydrogenase
mOS: median overall survival
mt: mutant
OS: overall survival
*TERT*p: telomerase reverse transcriptase promoter
wt: wildtype

## 1 Introduction

Glioma, a prevalent central nervous system (CNS) tumor, accounts for approximately 24% of all primary brain and CNS tumors, with 80.9% of them being malignant [1]. Among gliomas, glioblastoma (GBM) is the most fatal subtype, with a mere 5‐year survival rate of approximately 6.9%, and it constitutes 59.2% of all gliomas and has an incidence of 3.26 per 100,000 population [1]. In addition, CNS tumors are also frequent solid tumors in adolescents [2], and pediatric gliomas represent an age‐adjusted incidence rate of about 2.91 per 100,000 population [3]. For both adult‐type and pediatric‐type gliomas, since the 2016 World Health Organization (WHO) classification of CNS tumors, molecular profiling has become an indispensable step in diagnosis, and the updated 2021 WHO CNS Tumors Classification (referred to as WHO CNS5) continually strength the role molecular profiling plays [4, 5]. Nowadays based on WHO CNS5, molecular profiles and histological manifestations are used together to confirm tumor subtype and tumor grade. The prognosis of gliomas is significantly affected by these molecular alterations, such as whether *IDH1, IDH2, EGFR, CDKN2A*, and *CDKN2B* are altered for adult‐type gliomas, and whether H3 K27 and H3 G34 are altered for pediatric‐type gliomas [4].

Telomeres play a crucial role in limiting cell proliferation by shortening with each cell cycle, ultimately leading to apoptosis when they become too short to stabilize chromosomes. Telomerase reverse transcriptase (*TERT*) is the catalytic protein subunit of telomerase, responsible for elongating and stabilizing telomeres [6]. *TERT* transcription is consistently suppressed in somatic cells, but *TERT* promoter (*TERT*p) mutations have been identified in various cancers, including gliomas [7, 8]. These mutations, specifically cytidine‐to‐thymidine transitions (C228T and C250T), generate de novo binding sites for certain transcription factors, such as GA‐binding protein (GABP) or E‐twenty‐six (ETS) transcription factors, resulting in increased *TERT* expression and telomerase activity [8, 9, 10]. *TERT*p mutations are common in gliomas, occurring in around 40% of Grade II and III gliomas, 40% of IDH‐mutant (IDH‐mt) gliomas, and 70% of IDH‐wildtype (IDH‐wt) gliomas [11, 12, 13, 14].

The presence of *TERT*p mutation (*TERT*p‐mt) has significant implications for the prognosis of gliomas. Studies have demonstrated that *TERT*p mutation predicts a favorable prognosis in the IDH‐mt glioma subgroup while indicating a poor prognosis in the IDH‐wt subgroup [13, 15, 16]. Univariate analysis showed that the presence of *TERT*p mutation was associated with better overall survival (OS) in oligodendroglioma and was related to worse OS in IDH‐wt astrocytoma [17]. Conversely, patients with *TERT*p mutation had significantly decreased OS in histologic glioblastoma (GBM), regardless of IDH‐mt or IDH‐wt status [17]. Additionally, some research has suggested that *TERT*p‐mt predicts a worse prognosis in primary GBM [18].

Molecular changes have become valuable in predicting outcomes and improving the classification of gliomas. The 2016 WHO CNS Tumors Classification incorporated molecular markers for the first time. In the updated WHO CNS5, more molecular markers were included, with three genetic features (*TERT*p mutation, *EGFR* gene amplification, [+7/−10] combined gain of entire chromosome 7 and loss of chromosome 10) becoming the criteria for diagnosing IDH‐wt GBM [4, 5].

Compared to the 2016 classification, the definitions of astrocytoma and GBM have significantly changed, and neoplasms are now graded within types under the 2021 classification. Moreover, previous research indicated that the new criteria resulted in around 30% of low‐grade gliomas being reclassified as high‐grade gliomas. Furthermore, some cases have been categorized as pediatric types, and molecular GBMs have emerged [19]. The updated WHO guidelines place greater emphasis on the role of *TERT*p in GBM, but its applicability to IDH‐mt gliomas has not been strongly emphasized. Therefore, the validity of some existing conclusions about *TERT*p mutation remains unknown. It is essential to reevaluate the incidence and distribution of *TERT*p mutation and its impact on prognosis in different subtypes and subgroups under the new classification, as this aspect has not been thoroughly investigated. This study aims to fill this gap and additionally compare the prognosis between molecular GBM and histologic GBM, evaluate the impact of *TERT*p mutation and *EGFR* amplification on the prognosis of adult diffuse glioma, and explore the relationship between *TERT*p mutation and other molecular alterations, providing further insights into the use of *TERT*p within the context of the 2021 classification.

## 2 Material and Methods

### 2.1 Study Participants

The study included all glioma patients admitted to Peking Union Medical College Hospital and who underwent surgical resection or biopsy between January 2011 and April 2022. Patients without accessible formalin‐fixed paraffin‐embedded (FFPE) tumor tissue sections were excluded.

Ethical approval for this study was obtained from the Institutional Ethics Review Board of Peking Union Medical College Hospital (S‐424). Written informed consent was obtained from every subject.

### 2.2 Data Acquisition

Clinical information such as age, sex, body mass index (BMI), and preoperative Karnofsky Performance Status (KPS) was collected and analyzed. Overall survival (OS) was defined as the time from surgical resection or biopsy to death or the last follow‐up date if the patient was still alive. Survival rates at 1 year (1y‐OS) and 5 years (5y‐OS) were calculated for patients with OS over 1 and 5 years, respectively. Patients lost to follow‐up were censored at the date of their last contact. Patients who died from causes unrelated to glioma were also censored at their date of death. Patients still alive at the end of the study period were censored at their last known follow‐up date. Preoperative MRI profiles were reviewed by experienced neuroradiologists to identify image characteristics, including site, location, functional area invasion, contrast enhancement, tumor diameter, edema, and necrosis.

### 2.3 Gene Detection

DNA extraction from all FFPE tumor tissue sections was performed using the QIAGEN 56404 kit. Library preparation, fluorescence in situ hybridization (FISH), and PCR amplification were conducted following established methodologies [20]. Paired‐end sequencing was performed on the NovaSeq 6000 platform. SNP and CNV variations were analyzed using previously described approaches [21, 22]. A total of 60 molecular markers potentially affecting prognosis were screened based on recent research results and the WHO CNS5 classification [19]. *TERT*p mutation was detected for the C228T and C250T sites only.

### 2.4 Glioma Classification

Based on sequencing results and histological appearance under a microscope, all tumor samples were classified according to the WHO CNS5 classification. For IDH‐mutant gliomas, we categorize them into oligodendrogliomas and astrocytomas based on the presence or absence of 1p/19q codeletion. As for IDH wild‐type gliomas, when they are H3 wildtype, we differentiate them into glioblastoma (GBM) based on the presence of one or more of the following features: microvascular proliferation, intratumoral necrosis, *TERT* promoter mutation, *EGFR* amplification, or + 7/−10 mutations. Otherwise, they are classified as other subtypes of gliomas according to their molecular characteristics, such as *MYB* or *MYBL1* alterations or H3 gene mutations.

### 2.5 Statistical Analysis

Pairwise Fisher's test was used to analyze the correlation of different gene alterations in R software (version 4.2.1). Odds ratios of gene pairs were visualized using GraphPad Prism (version 8.3.0). Survival analysis was conducted using the Kaplan–Meier method with the log‐rank test. To eliminate the effect of confounders, patients were divided into subgroups based on certain characteristics, and Kaplan–Meier survival analysis was used within subgroups. Median OS (mOS) and 95% confidence interval (95% CI) were calculated for each subgroup. Univariate Cox proportional hazards regression analysis with hazard ratios (HRs) and 95% confidence intervals (CIs) was also used to assess the association with OS. These statistical analyses were performed using R software (version 4.2.1) and SPSS statistical analysis software v26 (IBM Corp., New York, NY, USA). A *p* value less than 0.05 was considered statistically significant.

## 3 Results

### 3.1 Distribution of *TERT* Promoter Mutation

**FIGURE 1:**
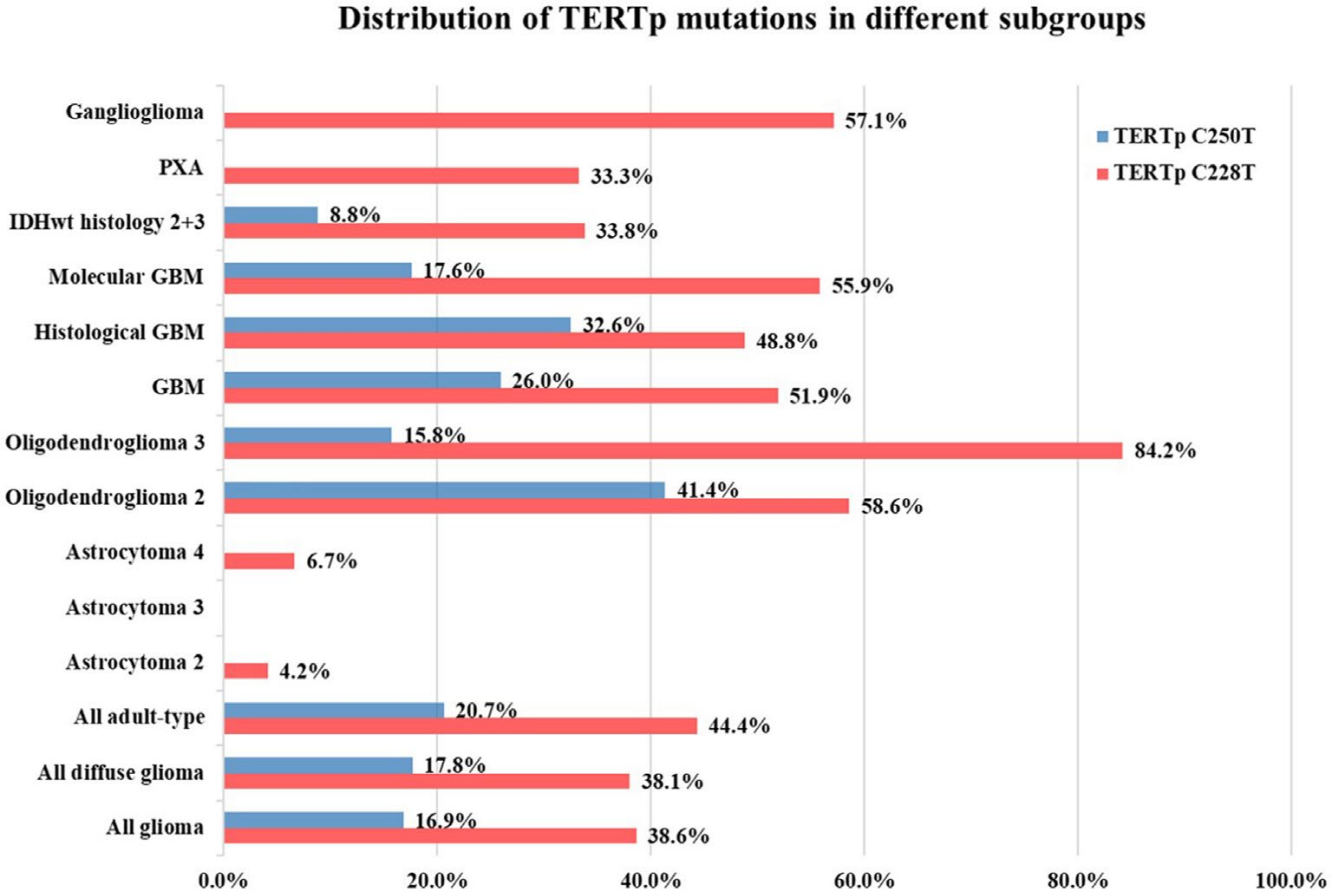
Distribution of *TERT*p mutation in different subgroups.

A total of 207 glioma patients were included. 169 patients were classified as patients with adult‐type gliomas, with 146 individuals remaining in the follow‐up cohort at the study's conclusion. Among the follow‐up cohort, 35 were astrocytoma, 69 were glioblastoma, and 42 were oligodendroglioma. The basic information, clinical features, and imaging information of 169 adult‐type glioma patients are summarized in Table 1. The term “*TERT*p mutations” described in the rest of this article include both C228T and C250T, unless specifically stated as C228 or C250 mutations. Among adult‐type gliomas, 2 out of 44 (4.55%) astrocytomas, 48 (100%) oligodendrogliomas, and 60 out of 77 (77.92%) GBMs carried *TERT*p mutations. In three cases of pleomorphic xanthoastrocytoma (PXA), 1 *TERT*p mutations were observed, and were present in four out of seven cases of gangliogliomas. Overall, there were 80 (38.6%) C228T and 35 (16.9%) C250T mutations. The detailed distribution of *TERT*p mutations in different subtypes or subgroups is shown in Figure 1.

**TABLE 1:** Basic information, clinical features, and imaging information of patients with adult‐type diffuse glioma.

	Astrocytoma, IDH‐mutant		Oligodendroglioma, IDH‐mutant, and 1p/19q‐codeleted	Glioblastoma, IDH‐wildtype	
	*TERT*p mutant	*TERT*p wildtype	*TERT*p mutant	*TERT*p mutant	*TERT*p wildtype
Number	2 (4.55%)	42 (95.45%)	48 (100%)	60 (77.92%)	17 (23.08%)
Gender					
Male	2 (100%)	27 (64.29%)	33 (68.75%)	41 (68.33%)	7 (41.18%)
Female	0	15 (35.71%)	15 (31.25%)	19 (31.67%)	10 (58.82%)
Mean age, year	45.00 ± 1.41	41.36 ± 11.00	43.14 ± 10.21	56.53 ± 13.38	48.71 ± 22.01
Age, year					
< 18	0	0	0	0	1
18–44	1	30	21	9	5
45–64	1	10	27	30	7
≥ 65	0	2	0	21	4
Mean BMI, kg/m^2^	22.00 ± 2.00	24.43 ± 2.95	25.17 ± 3.53	23.69 ± 2.97	23.72 ± 2.97
BMI, kg/m^2^					
< 18	0	0	0	1	0
18–24	2	18	18	31	10
> 24	0	23	29	26	6
Preoperative KPS	95.00 (92.5, 97.5)	82.50 (80, 100)	92.60 (90, 100)	81.58 (78.75, 100)	85.31 (70, 100)
Intracranial hypertension	0	24 (57.14%)	17 (35.42%)	27 (45%)	6 (35.29%)
Neurologic impairment	0	22 (52.38%)	22 (45.83%)	40 (66.67%)	10 (58.82%)
Epilepsy	1 (50%)	15 (35.71%)	23 (47.92%)	20 (33.33%)	4 (23.53%)
Histological grade classification					
WHO grade 2	1 (50%)	23 (54.76%)	29 (60.42%)	NA	NA
WHO grade 3	0	5 (11.90%)	19 (39.58%)	NA	NA
WHO grade 4	1 (50%)	14 (33.33%)	NA	60 (100%)	17 (100%)
Ki‐67, %	4.00 (3.5, 4.5)	12.32 (2, 10)	13.59 (3, 15)	30.61 (10, 50)	25.38 (2, 40)
Extent of surgical resection					
Total resection	0	21 (50.00%)	33 (68.75%)	38 (63.33%)	9 (52.94%)
Subtotal resection	2 (100%)	3 (7.14%)	2 (4.17%)	3 (5%)	1 (5.88%)
Partial resection	0	10 (23.81%)	3 (6.25%)	8 (13.33%)	2 (11.76%)
Biopsy	0	5 (11.90%)	6 (12.5%)	10 (16.67%)	3 (17.65%)
Side					
Right	1 (50%)	16 (38.10%)	19 (39.58%)	22 (36.67%)	4 (23.53%)
Left	1 (50%)	15 (35.71%)	21 (43.75%)	28 (46.67%)	8 (47.06%)
Bilateral	0	6 (14.29%)	5 (10.42%)	3 (5%)	2 (11.76%)
Location					
Parietal lobe	1 (50%)	3 (7.14%)	8 (16.67%)	13 (21.67%)	5 (29.41%)
Frontal lobe	2 (100%)	32 (76.19%)	33 (68.75%)	24 (40%)	8 (47.06%)
Temporal lobe	0	11 (26.19%)	12 (25%)	27 (45%)	4 (23.53%)
Occipital lobe	0	1 (2.38%)	2 (4.17%)	3 (5%)	2 (11.76%)
Insula	1 (50%)	5 (11.90%)	9 (18.75%)	7 (11.67%)	1 (5.88%)
Corpus callosum	0	3 (7.14%)	3 (6.25%)	4 (6.67%)	1 (5.88%)
Functional area	2 (100%)	9 (21.43%)	10 (20.83%)	29 (48.33%)	6 (35.29%)
Contrast enhancement	2 (100%)	14 (33.33%)	18 (37.5%)	46 (76.67%)	10 (58.82%)
Maximum diameter of tumor, cm	6.55 (6.02, 7.08)	6.10 (3.30, 5.84)	4.73 (3.00, 6.43)	3.89 (2.38, 5.00)	3.62 (2.44, 4.58)
Edema diameter, cm	0.95 (0.72, 1.18)	1.10 (0.00, 2.00)	1.13 (0.00, 2.00)	2.21 (0.90, 3.38)	1.90 (0.78, 2.77)
Necrosis diameter, cm	2.50 (2.05, 2.95)	0.79 (0.00, 1.30)	0.90 (0.00, 1.78)	1.85 (0.00, 3.00)	1.97 (0.00, 3.00)

Abbreviation: NA, not applicable.

Interestingly, only two patients with actrocytomas carried *TERT*p mutations, both exhibiting the C228T mutation, along with an *IDH1* mutation (p.R132H), but without co‐deletion of chromosome 1p/19q. Both gliomas exhibited mild enhancement on contrast‐enhanced MRI. Patient 1 had a homozygous deletion of *CDKN2A* and *CDKN2B*, a *ATRX* mutation of p.F2113fs, a *TP53* mutation of p.R273C, and exhibited dispersed anaplasia histologically, leading to a CNS WHO grade 4. Patient 2 had no deletion of *CDKN2A* or *CDKN2B*, a wildtype of *ATRX* and *TP53* gene, and exhibited well differentiated glioma without anaplasia, leading to a CNS WHO grade 2.

### 3.2 Prognostic Value of *TERT* Promoter Mutation in Adult‐Type Diffuse Gliomas

**FIGURE 2:**
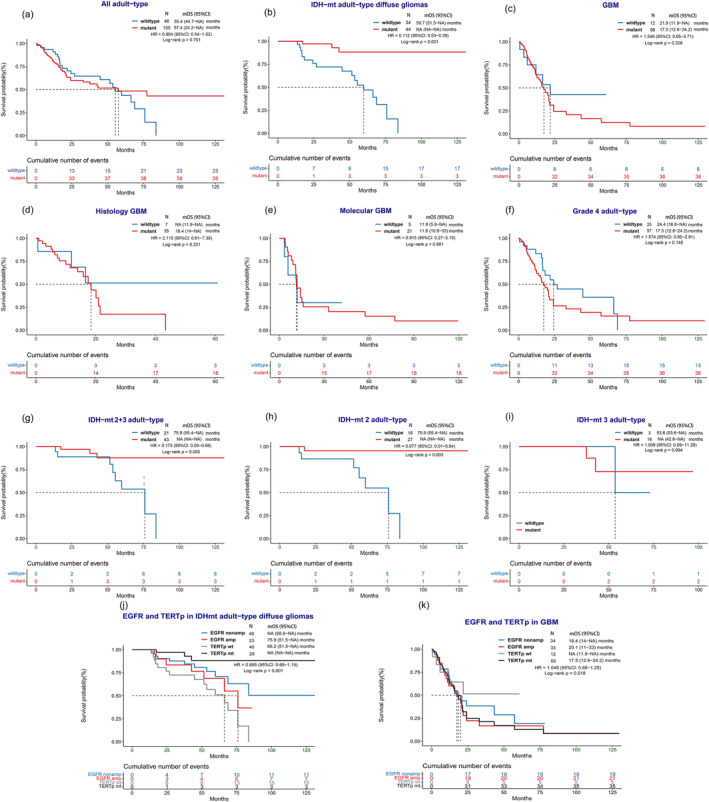
Overall survival of *TERT* promoter mutation in different subgroup of adult‐type diffuse glioma. (a) In all adult‐type gliomas. (b) In IDH mutant adult‐type diffuse gliomas. (c) In IDH wildtype glioblastomas. (d, e) In histology GBM and molecular GBM. (f) In all Grade 4 adult‐type gliomas including both IDH wildtype and mutant. (g) In IDH mutant Grades 2 and 3 gliomas. (h, i) In IDH mutant grade 2 or grade 3 adult‐type gliomas, respectively. (j) *EGFR* amplification and *TERT*p status in IDHmt adult‐type diffuse gliomas. (k) *EGFR* amplification and *TERT*p status in GBM. (l) *CDKN2A/B* Homozygous Deletions and *TERT*p status in IDHmt adult‐type diffuse gliomas. (m) *CDKN2A/B* Homozygous Deletions and *TERT*p status in GBM. IDHmt, IDH mutant. mOS, median OS. GBM, glioblastoma.

When assessing all adult‐type diffuse gliomas, *TERT*p mutation did not exhibit a significant difference in survival (median OS: 57.4 (95% CI: 24.2–not reached (NA)) vs. 55.4 (95% CI: 44.7–NA) months, HR = 0.904 (95% CI: 0.54–1.52), *p* = 0.701) (Figure 2a). However, in the IDH‐mutant (IDH‐mt) subgroup, *TERT*p mutations were associated with better survival (median OS: not reach [NA] (95% CI: NA–NA) vs. 59.7 (95% CI: 51.5–NA) months, HR = 0.111 (95% CI: 0.03–0.39), *p* < 0.001) (Figure 2b). This survival benefit was not observed in the IDH‐wildtype (IDH‐wt) GBM subgroup (median OS: 17.5 (95% CI: 12.6–24.2) vs. 21.9 (95% CI: 11.9–NA) months, HR = 1.677 (95% CI: 0.7–4.01), *p* = 0.242) (Figure 2c).

Further subgroup analyses were conducted based on grade and genetic background. Histologic GBM was defined as IDH‐wt GBM with histological grade 4 features, with or without molecular alterations. Molecular GBM was classified as GBM with histological grade 2 or 3 and at least one of the molecular alterations, including *TERT*p mutation, *EGFR* amplification, and + 7/−10 copy number changes. No significant difference in prognosis was observed between these two subgroups (median OS: 18.4 (95% CI: 15.8–NA) vs. 16.1 (95% CI: 11.4–33) months, HR = 1.07 (95% CI: 0.57–2), *p* = 0.839) (Figure S1). *TERT*p mutation was not associated with survival in histologic GBM (median OS: 18.4 (95% CI: 14–NA) vs. NA (95% CI: 11.9–NA) months, HR = 2.115 (95% CI: 0.61–7.39), *p* = 0.231) or molecular GBM (median OS: 15.2 (95% CI: 11–57.4) vs. 21.9 (95% CI: 5.8–NA) months, HR = 1.232 (95% CI: 0.36–4.24), *p* = 0.746) (Figure 2d,e). Similar results were observed when considering all Grade 4 adult‐type gliomas (median OS: 17.5 (95% CI: 12.6–24.2) vs. 24.4 (95% CI: 16.8–NA) months, HR = 1.574 (95% CI: 0.85–2.91), *p* = 0.145) (Figure 2f). However, for Grade 2 + 3 IDH‐mt diffuse gliomas, *TERT*p mutation was associated with a better prognosis (median OS: NA (95% CI: NA–NA) vs. 75.9 (95% CI: 55.4–NA) months, HR = 0.175 (95% CI: 0.05–0.68) *p* = 0.005) (Figure 2g). The same conclusion was validated in Grade 2 IDH‐mutant diffuse gliomas (median OS: NA (95% CI: NA–NA) vs. 75.9 (95% CI: 55.4–NA) months, HR = 0.077 (95% CI: 0.01–0.64), *p* = 0.003) (Figure 2h), but it was not observed in grade 3 gliomas (median OS: NA (95% CI: 42.6–NA) vs. 53.6 (95% CI: 53.6–NA) months, HR = 1.009 (95% CI: 0.09–11.29), *p* = 0.994) (Figure 2i), possibly due to the limitation of the sample size in the Grade 3 glioma cohort.

Interestingly, previous studies have shown that C228T and C250T mutations in the *TERT* promoter may have different molecular mechanisms and varying impacts on the prognosis of glioma patients [23]. However, in this study, a prognostic difference was only observed in histologic GBM (median OS: 15.8 (95% CI: 7.8–NA) vs. 21 (95% CI: 20.1–NA) months, HR = 0.326 (95% CI: 0.11–0.96), *p* = 0.033), while no significant differences were found in other subgroups (Figure S2).

Moreover, in the 2021 WHO classification, *EGFR* is considered equally important as a characteristic molecular marker alongside *TERT*. To assess its prognostic significance, our study compared the survival rates between *EGFR* amplification and *TERT*p mutation. The results demonstrated that in IDH‐mt adult‐type diffuse glioma patients, *TERT* promoter mutations were significantly associated with a better prognosis as same to *EGFR* non‐amplified (HR = 0.933 (95% CI: 0.78–1.12), *p* = 0.001). Conversely, wildtype *TERT* promoter was correlated with the poorest survival rates. However, in GBM, there appeared to be less clear distinction in prognostic significance based on these markers (HR = 1.021 (95% CI: 0.9–1.16), *p* = 0.624) (Figure 2j,k).

In order to further investigate the association between these two molecules, we extended our analysis to examine the impact of *TERT*p on prognosis under different *EGFR* conditions. Similar to adult‐type gliomas overall, we have observed that the influence of the *TERT* promoter on prognosis remains consistent across different *EGFR* statuses. In adult‐type gliomas with an IDH mutation, *TERT* promoter mutation correlates with a better prognosis; while this distinction is not evident in the context of GBM (Figure S3).

*CDKN2A/B* is also a signature molecule in IDH‐mt adult‐type diffuse gliomas. To evaluate its prognostic significance, further survival comparison was made between *CDKN2A/B* mutation and *TERT*p mutation. The results showed that in IDH‐mt adult‐type diffuse glioma patients, *CDKN2A/B* wildtype or *TERT* promoter mutation was associated with a remarkably better prognosis (HR = 0.974 (95% CI: 0.82–1.16), *p* < 0.001), while *TERT*p wildtype was associated with the worst survival, while the similar results were not observed (HR = 1.02 (95% CI: 0.9–1.16), *p* = 0.662) (Figure 2l,m). Similarly, the favor prognosis of *TERT* promoter mutation in *CDKN2A/B* none homozygous deletion IDH mutant adult‐type gliomas, which the difference with the *CDKN2A/B* homozygous deletion group may be caused by the limited number of *CDKN2A/B* homozygous deletion group. Identically, *TERT* promoter did not exhibit prognosis value in GBM no matter *CDKN2A/B* status (Figure S4).

We validated the results of Kaplan–Meier survival analysis in Figure 2 with univariate COX regression analysis, obtaining the same effect of these molecular alterations on survival, which are displayed in Data S6.

### 3.3 Contributions of *TERT* Promoter Mutations to Other Glioma Types

**FIGURE 3:**
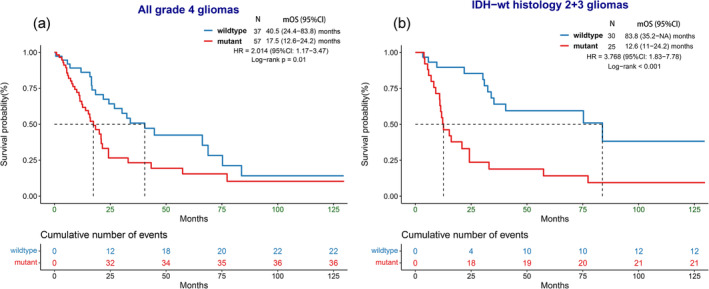
Overall survival of *TERT* promoter mutation in other subtype combinations. (a) In all grade 4 gliomas, including pediatric type. (b) In IDH wildtype glioma with histological grade 2 and 3 appearance. IDHwt, IDH wildtype. mOS, median OS.

In addition to adult‐type glioma, the study analyzed the prognostic impact of *TERT*p mutation in all gliomas, including pediatric gliomas such as diffuse midline glioma and pediatric‐type high‐grade glioma. Unlike adult‐type grade 4 gliomas, *TERT*p mutation was associated with poor OS in all Grade 4 gliomas (median OS: 17.5 (95% CI: 12.6–24.2) vs. 40.5 (95% CI: 24.4–83.8) months, HR = 2.014 (95% CI: 1.17–3.47), *p* = 0.01) (Figure 3a).

Furthermore, the impact of *TERT*p mutation status on IDH‐wt gliomas with Grades 2 and 3 histology (referred to as IDH‐wt histology grade 2 + 3) was analyzed. These gliomas included adult‐type diffuse gliomas, pediatric‐type diffuse gliomas, and circumscribed astrocytic gliomas. The mutation group in the IDH‐wt histology grade 2 + 3 subgroup showed significantly reduced OS (median OS: 12.6 (95% CI: 11–24.2) vs. 83.8 (95% CI: 35.2–NA) months, HR = 3.768 (95% CI: 1.83–7.78), *p* < 0.001) (Figure 3b). Similarly, the impact of C228T and C250T mutations on prognosis was also investigated, but no significant associations were observed in these cohorts with respect to survival outcomes.

### 3.4 Correlation Between *TERT* Promoter Mutation and Other Gene Alterations

**FIGURE 4:**
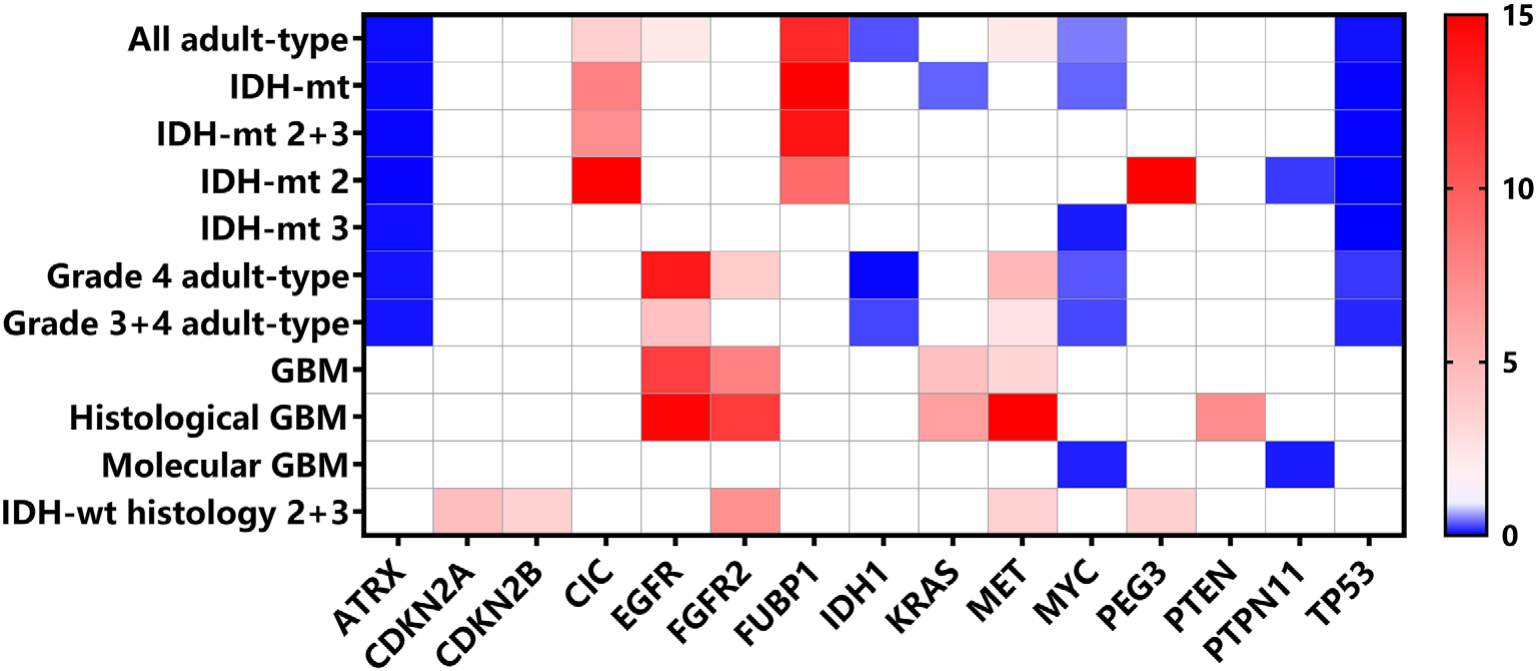
Correlation between *TERT*p mutation and other gene alterations. Odds ratio was used to show correlation between paired genes. Paired genes with *p* < 0.05 in the pairwise fisher test were indicated in red (co‐occurrence) or blue (exclusivity). Paired genes without significance were indicated in white. Only genes with at least one significant correlation were shown here. To better demonstrate the strength of the correlation, odds ratio values greater than 15 were denoted by 15. IDH‐mt, IDH mutant. IDH‐wt, IDH wildtype. GBM, glioblastoma.

In light of the prognostic impact of *TERT*p mutation, our study delved into the correlation between *TERT*p and a selected set of genes to identify subgroups with more profound prognostic significance (Figure 4).

In the IDH‐mutant subgroup, we observed a tendency for *TERT*p mutation to co‐occur with *CIC* and *FUBP1* alterations, while being mutually exclusive with *ATRX* and *TP53* alterations. These patterns were consistent in both IDH‐mutant grade 2 + 3 glioma and IDH‐mutant grade 2 glioma. However, the correlation was somewhat attenuated in the IDH‐mutant grade 3 subgroup.

Conversely, in the GBM subgroup, a different pattern emerged, where *TERT*p mutation tended to co‐occur with *EGFR, FGFR2, KRAS, and MET* alterations. Interestingly, when stratifying GBM into histologic GBM and molecular GBM, this correlation was amplified in histologic GBM and diminished in molecular GBM.

In Grade 3 and Grade 4 gliomas, regardless of IDH status, *TERT*p mutation displayed mutual exclusivity with *ATRX, TP53, IDH1*, and *MYC* alterations, while co‐occurring with *EGFR* and *MET* alterations.

Of particular note, in the IDH‐wt histology grade 2 + 3 subgroup, we found a molecular correlation more akin to GBM, distinct from the IDH‐mutant subgroup tumors.

Furthermore, we have explored the correlation between *TERT*p and several other prominent molecules, which can be observed in Figure S5. These findings provide valuable insights into the intricate molecular interactions involving *TERT*p mutation and other gene alterations. Understanding these correlations may hold the key to refining prognosis predictions and optimizing personalized treatment strategies for specific glioma subtypes.

## 4 Discussion

In this article, we have explored the distribution and prognostic significance of *TERT*p mutation, along with its molecular interactions with other gene alterations in various types of gliomas, following the latest WHO CNS5 classification. Our study revealed that *TERT*p‐mt was consistently present in all oligodendrogliomas, a majority of GBMs, but was notably scarce in astrocytomas. The prognostic value of *TERT*p mutation was predominantly observed in IDH‐mt glioma cases, whereas it did not exhibit significant prognostic implications in GBMs. However, *TERT*p mutation emerged as a robust marker distinguishing the survival outcomes of the IDH‐wt histologic grade 2 + 3 subgroup. Additionally, we conducted an in‐depth analysis to explore the correlation between *TERT*p mutation and other molecular alterations in gliomas.

In our study encompassing all adult‐type gliomas, *TERT* promoter mutations were observed in 65.1% of patients. Among these mutations, C228T was found to be twice as prevalent as C250T. This finding aligns with the conclusions drawn in a prior study, which highlighted the higher prevalence of C228T compared to C250T in adult‐type glioma [24]. Upon subgroup analysis, we discovered that 77.9% of GBM samples exhibited *TERT* promoter mutations, with the proportions of C228T and C250T mirroring those observed in the broader adult‐type glioma cohort. Notably, within IDH‐mt glioma cases, *TERT* promoter mutations were detected in all oligodendrogliomas but only in 4.55% of astrocytomas, consistent with the descriptions provided in the WHO CNS guidelines and supported by previous studies [25]. As for pediatric‐type gliomas, our study did not identify any *TERT* promoter mutations. Moreover, to the best of our knowledge, no previous studies have explored *TERT* promoter mutations specifically in the newly defined pediatric‐type glioma subgroup.

*TERT*p mutation has garnered significant attention due to its prognostic significance. Previous studies have indicated that *TERT*p‐mt is linked to shorter OS in GBM [23], while demonstrating a longer OS in Grades 2 and 3 gliomas based on earlier definitions [25]. In our current study, employing the latest WHO CNS5 classification, we did not observe a prognostic value for *TERT*p in GBM, histological GBM, molecular GBM, or Grade 4 adult‐type glioma. The prognostic significance of *TERT*p mutations in IDH‐wt GBM has not been well defined yet [26]. In addition, the three molecular criteria of the 2021 classification have upgraded approximately 30% of low‐grade gliomas to GBM [19]. The prognostic impact of *TERT*p mutations in such a newly constituted GBM population remains to be explored. The negative result of our research suggested that this GBM group may still be heterogeneous. The prognostic effect of *TERT*p mutations may be explored when subdividing this group by combining more molecular features, which warrants further investigation.

Furthermore, we noticed a divergent effect of *TERT*p mutation on adult‐type and all‐type gliomas. When examining all Grade 4 gliomas, which encompassed pediatric‐type gliomas with no *TERT*p mutation and slightly improved survival compared to adult‐type Grade 4 gliomas [27, 28], *TERT*p‐mt was associated with worse survival. This suggests that the prognostic value of *TERT*p mutation as a diagnostic marker has been attenuated within the GBM subgroup but remains notable in all Grade 4 gliomas. On the contrary, in lower grade gliomas, the prognostic value of *TERT*p mutation persists. In IDH‐mt gliomas, both with and without astrocytomas, Grade 4 patients with *TERT*p‐mt experienced significantly longer survival times, corroborating findings from previous research [29].

Interestingly, previous studies have suggested that C228T and C250T mutations may exert different impacts on patient prognosis, possibly due to distinct underlying mechanisms [24]. However, in our own research, we found that this differentiation was only evident within the histological GBM subgroup. This finding could have several implications. Firstly, it may indicate that the new classification methods employed possess higher prognostic predictive power and integrative capacity compared to previous approaches. Secondly, it suggests that, despite being categorized as GBM, further subgroups can be delineated within the GBM classification—namely, histological GBM and molecular GBM. The differences observed between these two subgroups warrant further investigation to gain a deeper understanding of their clinical implications and potential implications for patient management.

In addition to analyzing regular glioma subtypes, we endeavored to explore the behavior of tumors within certain combined subsets. Specifically, within the IDH‐wt histologic grade 2 + 3 subgroup, we observed that *TERT*p‐mt was significantly associated with a poor prognosis. This finding strongly supports the current molecular GBM definition, underlining the relevance and importance of *TERT*p mutation in delineating prognostic implications within this specific subgroup of gliomas.

Moreover, as both *TERT*p mutation and *EGFR* amplification are molecular criteria for GBM diagnosis, our aim was to explore the relationship between these two biomarkers. The results of our investigation revealed that these two biomarkers play distinct roles within their respective domains. Specifically, *TERT*p mutation and non‐amplified *EGFR* were associated with longer OS. Furthermore, *TERT*p mutation displayed a superior OS compared to non‐amplified *EGFR*, while wild‐type *TERT*p showed a worse OS in comparison to *EGFR* amplification. However, within the GBM subgroup, these differences were not observed. Furthermore, we have demonstrated that this prognostic effect of *TERT* promoter mutations holds true across different *EGFR* backgrounds. This suggests the possibility of additional molecular mechanisms that may influence the impact of these two markers on prognosis and tumor malignancy phenotype. Consequently, further research is warranted to thoroughly investigate these underlying mechanisms and their potential contribution to the observed differences in prognostic outcomes.

Additionally, we conducted analyses to determine the survival correlation between *TERT*p mutation and other molecular markers. Prior studies have demonstrated that in low‐grade IDH‐mutant gliomas, *TERT*p mutation is associated with genetic alterations in *CIC, FUBP1*, and *MYC*, as these genes are closely related to 1p/19q deletion [30, 31]. Conversely, astrocytomas are commonly associated with *ATRX* and p53 mutations [8, 24]. Our findings align with these previous conclusions, and we observed these correlations in low‐grade gliomas, while they were not evident in GBM cases.

In the GBM subgroups, we identified distinct molecular features in histologic GBM and molecular GBM. Histologic GBM exhibited associations with *EGFR*, *FGFR2, KRAS, MET*, and *PTEN*, whereas molecular GBM showed an inverse association with *MYC* and *PTPN11*. Previous research has indicated that *EGFR* amplification and *PTEN* deletion are notably more frequent in IDH‐wt *TERT*p mutant patients [32]. *PTEN* and *KRAS* mutations are considered crucial alterations for GBM pathogenesis through the activation of MAPK and PI3K signaling pathways [33]. *FGFR2* and *MET* mutations have been reto sustain GBM malignancy [34, 35]. The disparity in molecular patterns between histologic GBM and molecular GBM suggests that *TERT*p mutant GBM, with or without histological malignancy, differs in terms of oncogenesis, behavior, and even potential treatment approaches. These biomarkers warrant further study to evaluate their clinical value. It may be necessary to refine the classification and develop corresponding treatment strategies for GBM patients based on these distinct molecular subgroups.

However, it is important to acknowledge the limitations of our study. First, our patient cohort was relatively small, which may have limited the statistical power of certain analyses. In some subgroups, the number of patients was small, potentially affecting the robustness of our findings. Furthermore, our study included only patients for whom tumor tissue sections were available, which could introduce a selection bias. It is possible that patients without available tissue sections may have different molecular profiles or prognostic outcomes. Lastly, our detection method focused on a preselected genetic locus, which means that other molecular correlations may not have been fully accounted for. Future studies should consider comprehensive molecular profiling to capture a broader range of genetic alterations and their associations with prognosis in gliomas.

In conclusion, based on the fact that the entities included in each subtype have changed significantly compared to the 2016 classification [17], we reevaluated the prognostic significance of *TERT*p mutations in various subgroups in this research. The *TERT*p mutation demonstrated strong prognostic value in IDH‐mt adult‐type gliomas, all Grade 4 gliomas and IDH‐wt histology 3–3 gliomas, with the latter two cohorts encompassing not only adult‐type cases. Indeed, molecular correlations and *TERT*p mutation subtyping analysis suggested that certain classification methods could potentially be further divided into distinct subtypes. This observation highlighted the need for in‐depth research into the mechanisms involving *TERT*p in the occurrence and progression of gliomas. By elucidating the role of *TERT*p in glioma development and progression, we may uncover novel insights that could contribute to the advancement of diagnostic and therapeutic approaches for glioma patients.

## Author Contributions

**Hao Xing:** data curation (equal), resources (equal). **Delin Liu:** conceptualization (equal), supervision (equal). **Junlin Li:** formal analysis (equal), software (equal), writing – original draft (equal). **Yulu Ge:** data curation (equal), supervision (equal), writing – review and editing (equal). **Xiaopeng Guo:** data curation (equal), writing – review and editing (equal). **Wenlin Chen:** funding acquisition (equal), validation (equal), visualization (equal). **Dachun Zhao:** data curation (equal). **Yixin Shi:** resources (equal). **Yilin Li:** software (equal). **Yaning Wang:** supervision (equal). **Yuekun Wang:** validation (equal). **Yu Xia:** visualization (equal). **Jiaming Wu:** writing – original draft (equal). **Tingyu Liang:** writing – review and editing (equal). **Hai Wang:** project administration (equal). **Qianshu Liu:** methodology (equal). **Shanmu Jin:** investigation (equal). **Tian Qu:** formal analysis (equal). **Siying Guo:** data curation (equal). **Huanzhang Li:** data curation (equal). **Tianrui Yang:** data curation (equal). **Kun Zhang:** data curation (equal). **Yu Wang:** funding acquisition (equal). **Wenbin Ma:** data curation (equal).

## Ethics Statement

Approval of the research protocol by an Institutional Reviewer Board: This study involving human participants was reviewed and approved by the Ethics Committee of Peking Union Medical College Hospital.

## Conflicts of Interest

The authors declare no conflicts of interest.

## Supporting information


Data S1:

## Data Availability

The data that support the findings of this study are available from the corresponding author upon reasonable request.
