# Supplementary material for: TERT p Mutation and its Prognostic Value in Glioma Patients Under the 2021 WHO Classification: A Real‐World Study
Source: Cancer Med. 2025 Jan 13;14(2):e70533. doi: 10.1002/cam4.70533 (PMC11727134; doi:10.1002/cam4.70533)
Supplement: Supplementary file 1 — Data S1: [file CAM4-14-e70533-s001.zip › cam470533-sup-0005-FigureS5.docx]

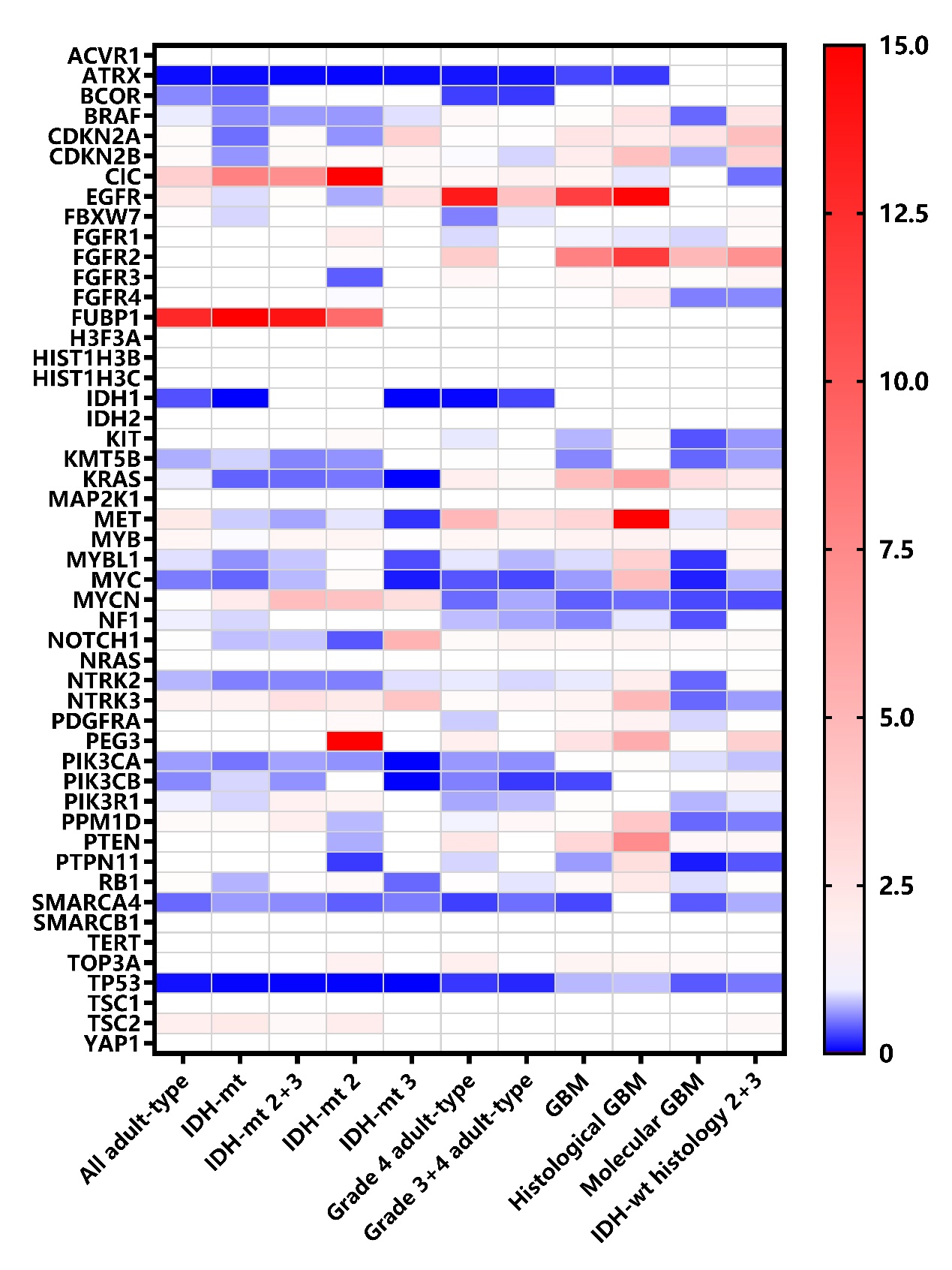


**Supplementary Figure 5. Correlation between TERTp mutation and other gene alterations.**

Odds ratio was used to show correlation between paired genes. Regardless of whether the P-value of pairwise fisher test was significant, the odd ratios of all computable gene pairs were displayed in this graph. Red showed co-occurrence and blue showed exclusivity. Paired genes whose odd ratio cannot be calculated were showed in white. To better demonstrate the strength of the correlation, odds ratio values greater than 15 were denoted by 15.
