# Supplementary material for: TERT p Mutation and its Prognostic Value in Glioma Patients Under the 2021 WHO Classification: A Real‐World Study
Source: Cancer Med. 2025 Jan 13;14(2):e70533. doi: 10.1002/cam4.70533 (PMC11727134; doi:10.1002/cam4.70533)

a

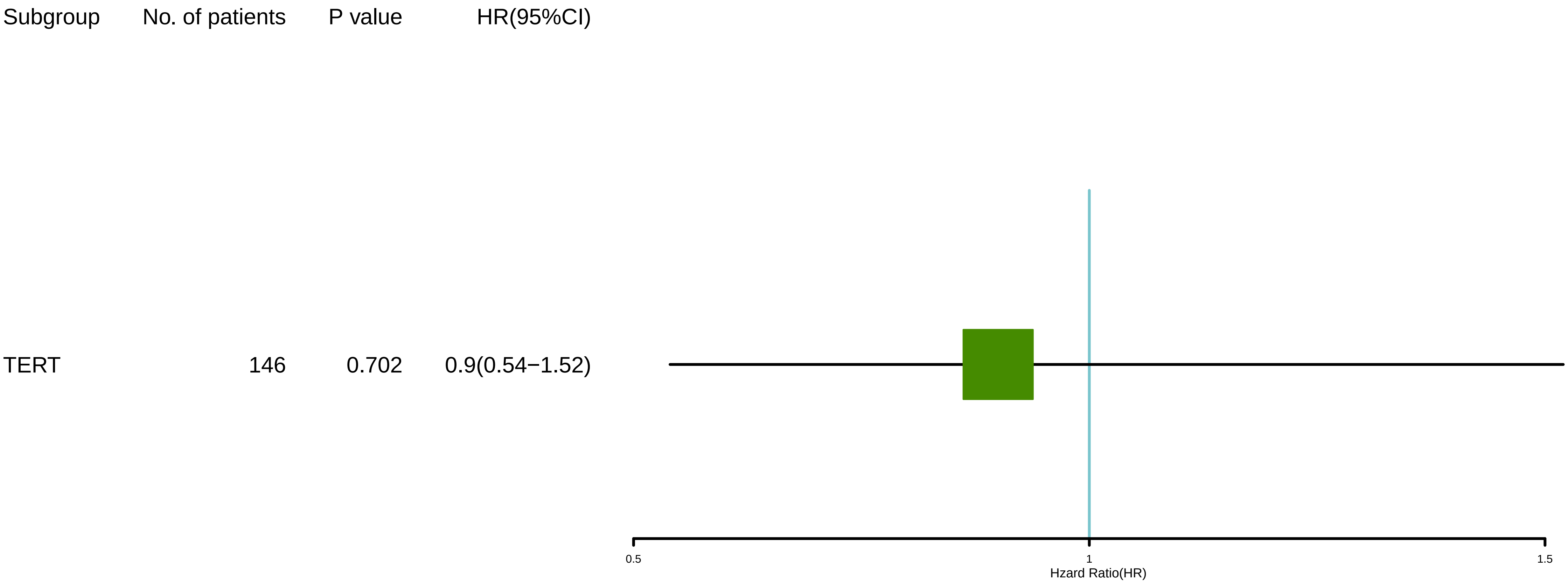
Subgroup
No. of patients
P value
HR(95%CI)
TERT
146
0.702
0.9(0.54−1.52)
0.5
1
1.5
Hzard Ratio(HR)

b

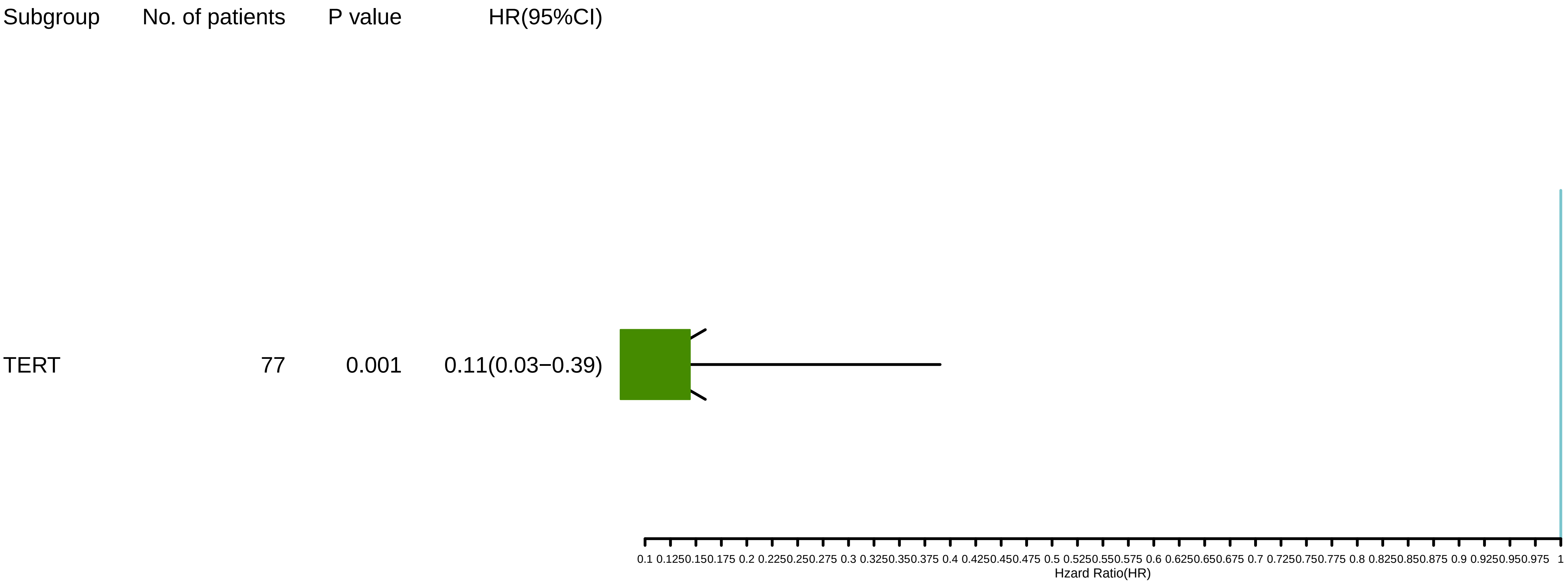

Subgroup
No. of patients
P value
HR(95%CI)
TERT
77
0.001
0.11(0.03−0.39)
0.1 0.125 0.15 0.175 0.2 0.225 0.25 0.275 0.3 0.325 0.35 0.375 0.4 0.425 0.45 0.475 0.5 0.525 0.55 0.575 0.6 0.625 0.65 0.675 0.7 0.725 0.75 0.775 0.8 0.825 0.85 0.875 0.9 0.925 0.95 0.975 1
Hazard Ratio(HR)

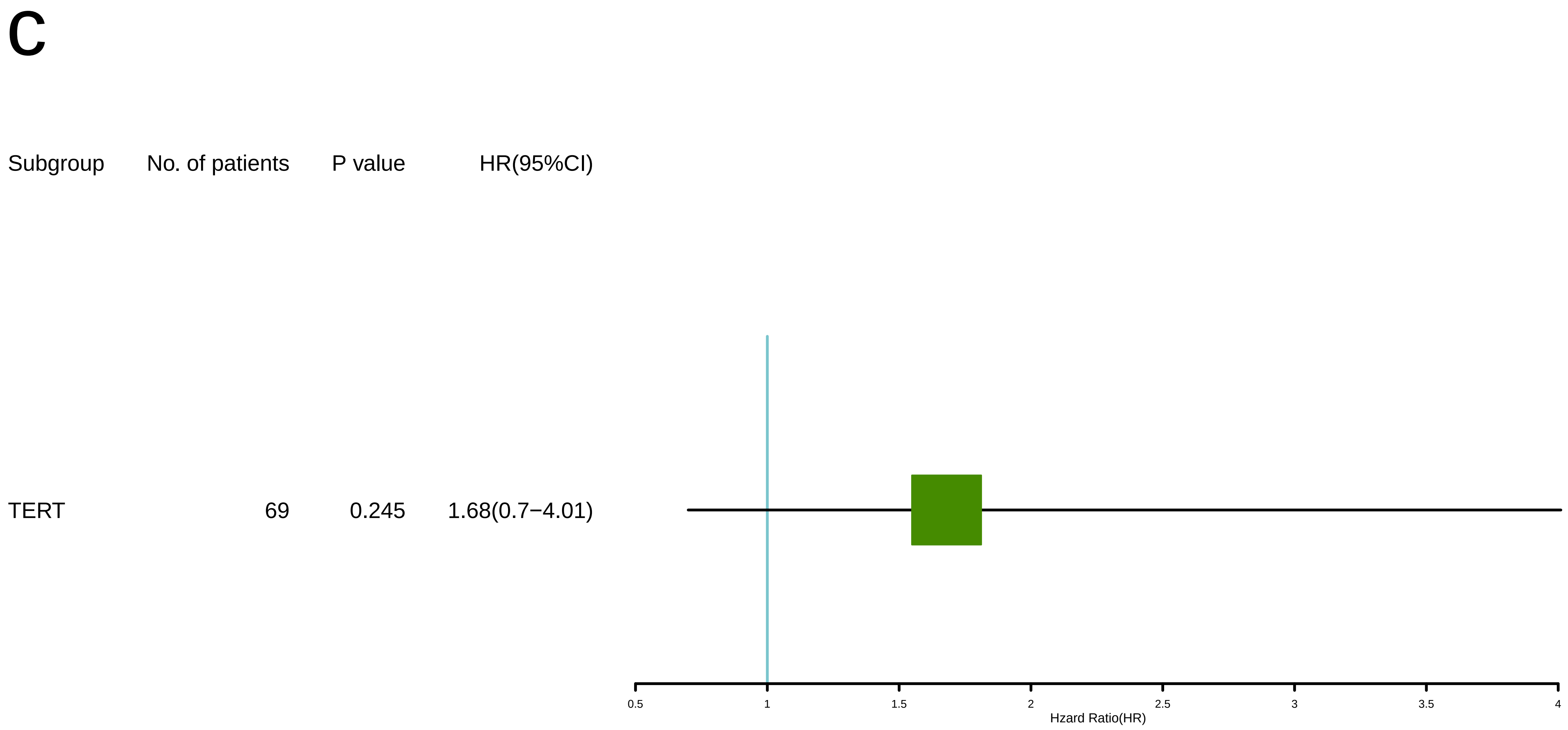
C
Subgroup
No. of patients
P value
HR(95%CI)
TERT
69
0.245
1.68(0.7−4.01)
0.5
1
1.5
2
2.5
3
3.5
4
Hzard Ratio(HR)

d

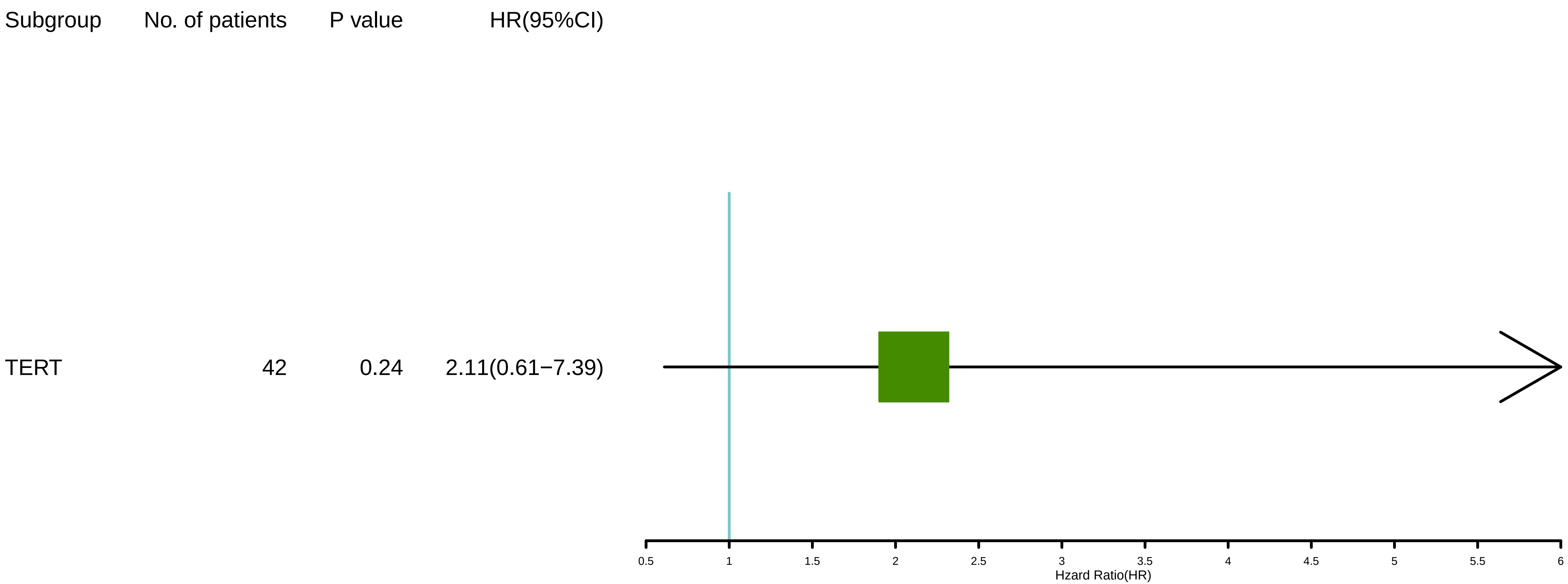
Subgroup
No. of patients
P value
HR(95%CI)
TERT
42
0.24
2.11(0.61−7.39)
0.5
1
1.5
2
2.5
3
3.5
4
4.5
5
5.5
6
Hzard Ratio(HR)

e

Subgroup	No. of patients	P value	HR(95%CI)
TERT	27	0.741	1.23(0.36−4.24)

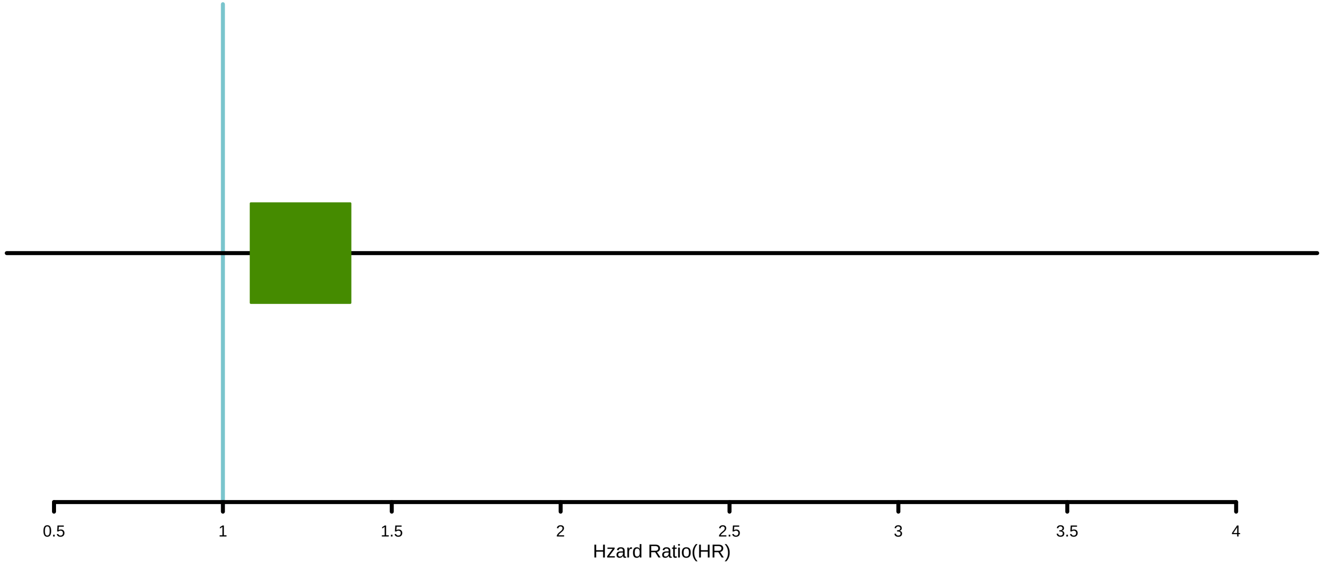

f

Subgroup	No. of patients	P value	HR(95%CI)
TERT	82	0.147	1.57(0.85−2.91)

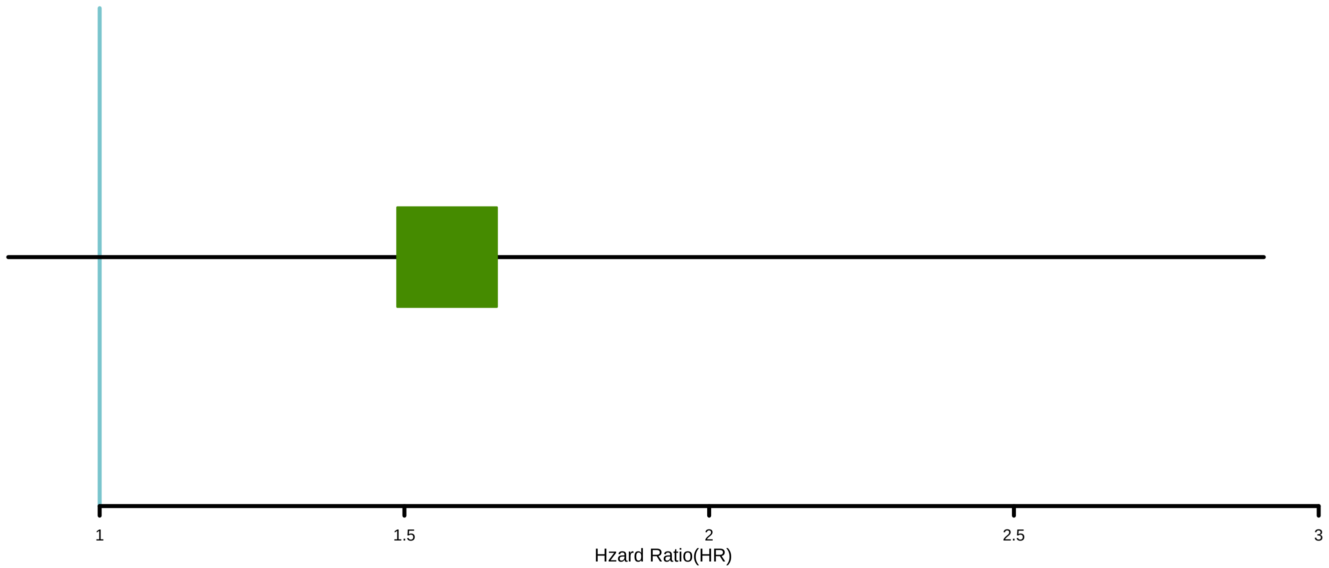

g

Subgroup	No. of patients	P value	HR(95%CI)
TERT	64	0.012	0.18(0.05−0.68)

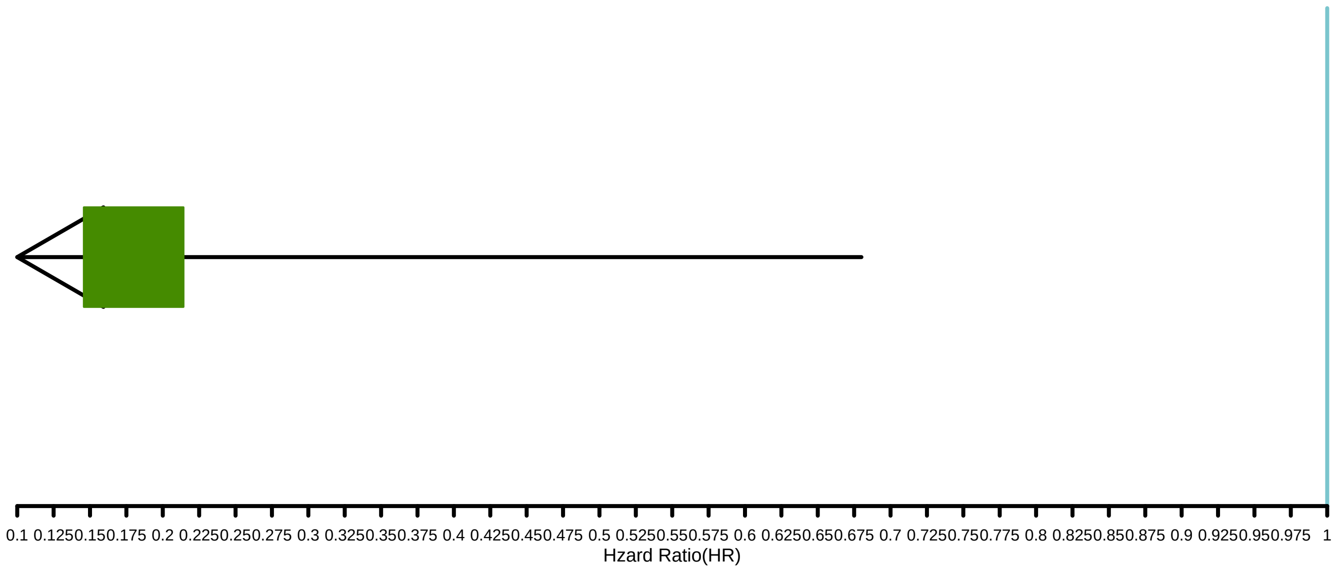

h

Subgroup	No. of patients	P value	HR(95%CI)
TERT	45	0.017	0.08(0.01−0.64)

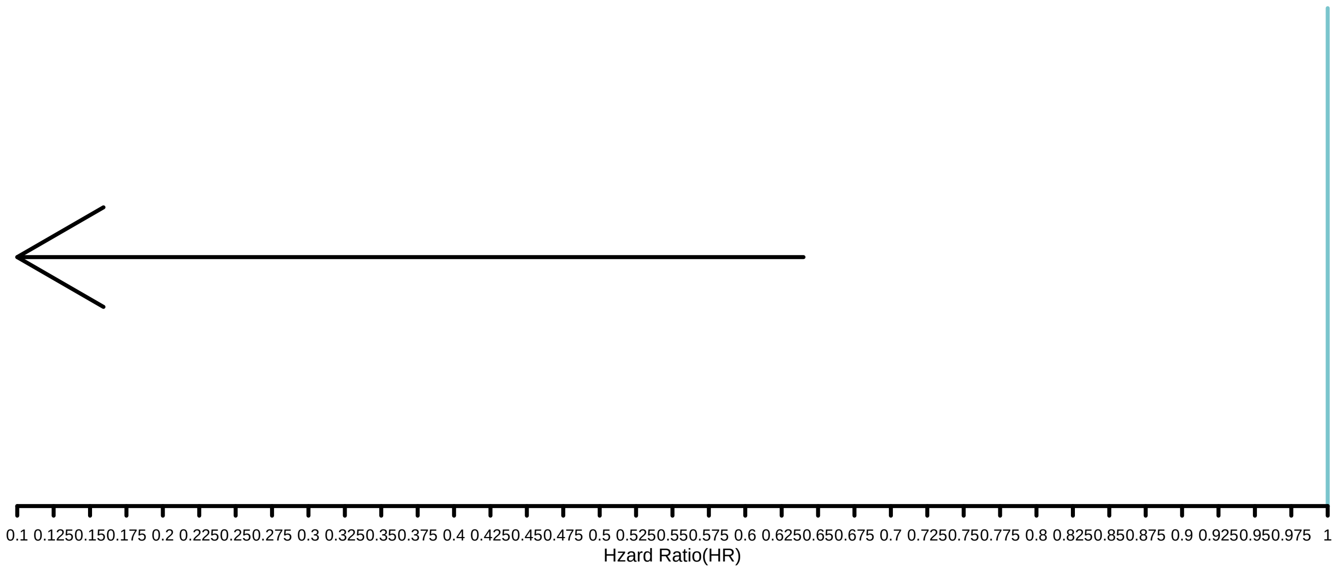

i

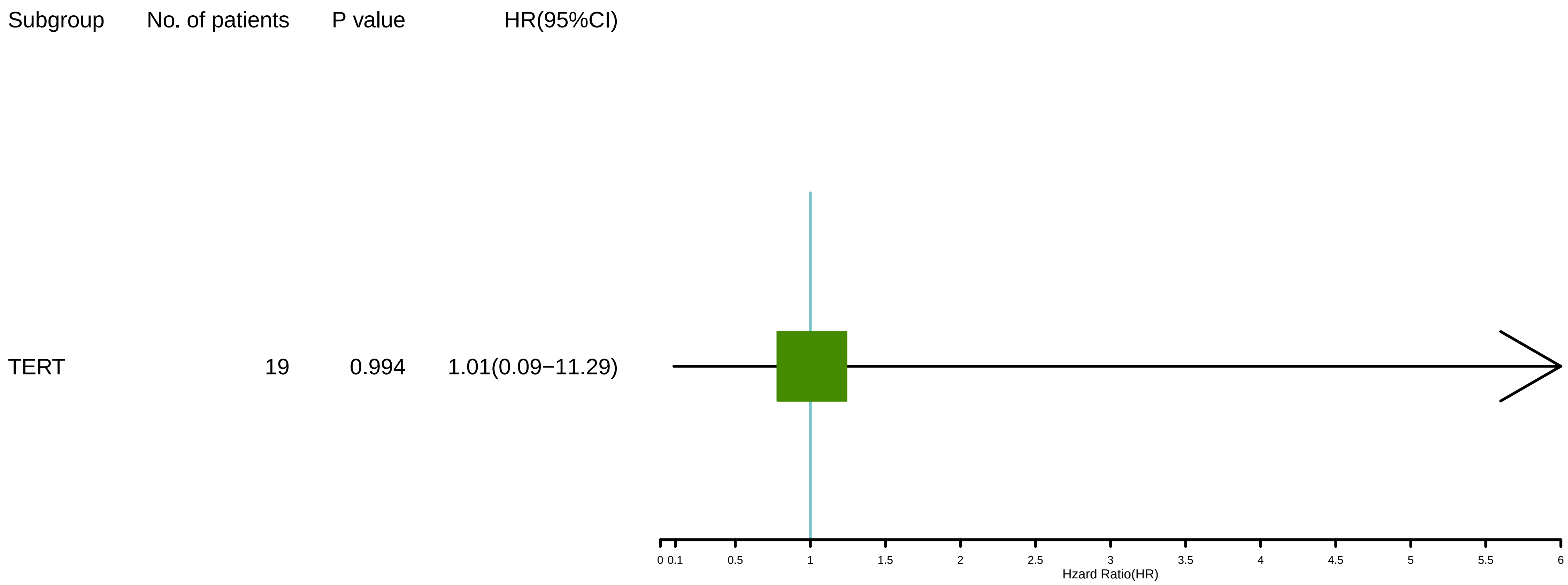

Subgroup
No. of patients
P value
HR(95%CI)
TERT
19
0.994
1.01(0.09−11.29)
0
0.1
0.5
1
1.5
2
2.5
3
3.5
4
4.5
5
5.5
6
Hzard Ratio(HR)

j

Subgroup	No. of patients	P value	HR(95%CI)
TERT	77	0.001	0.11(0.03−0.39)
EGFR	77	0.68	1.21(0.48−3.05)

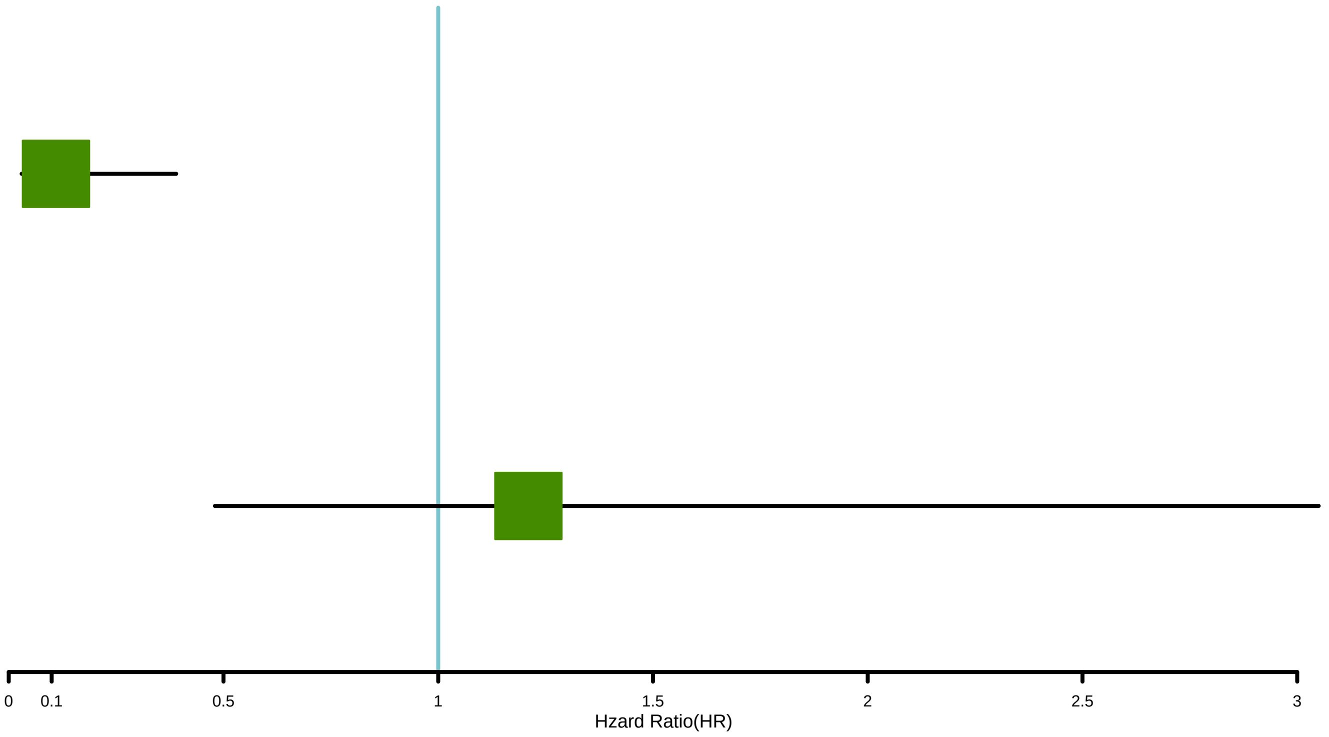

k

Subgroup	No. of patients	P value	HR(95%CI)
TERT	69	0.245	1.68(0.7−4.01)
EGFR	69	0.547	1.21(0.66−2.21)

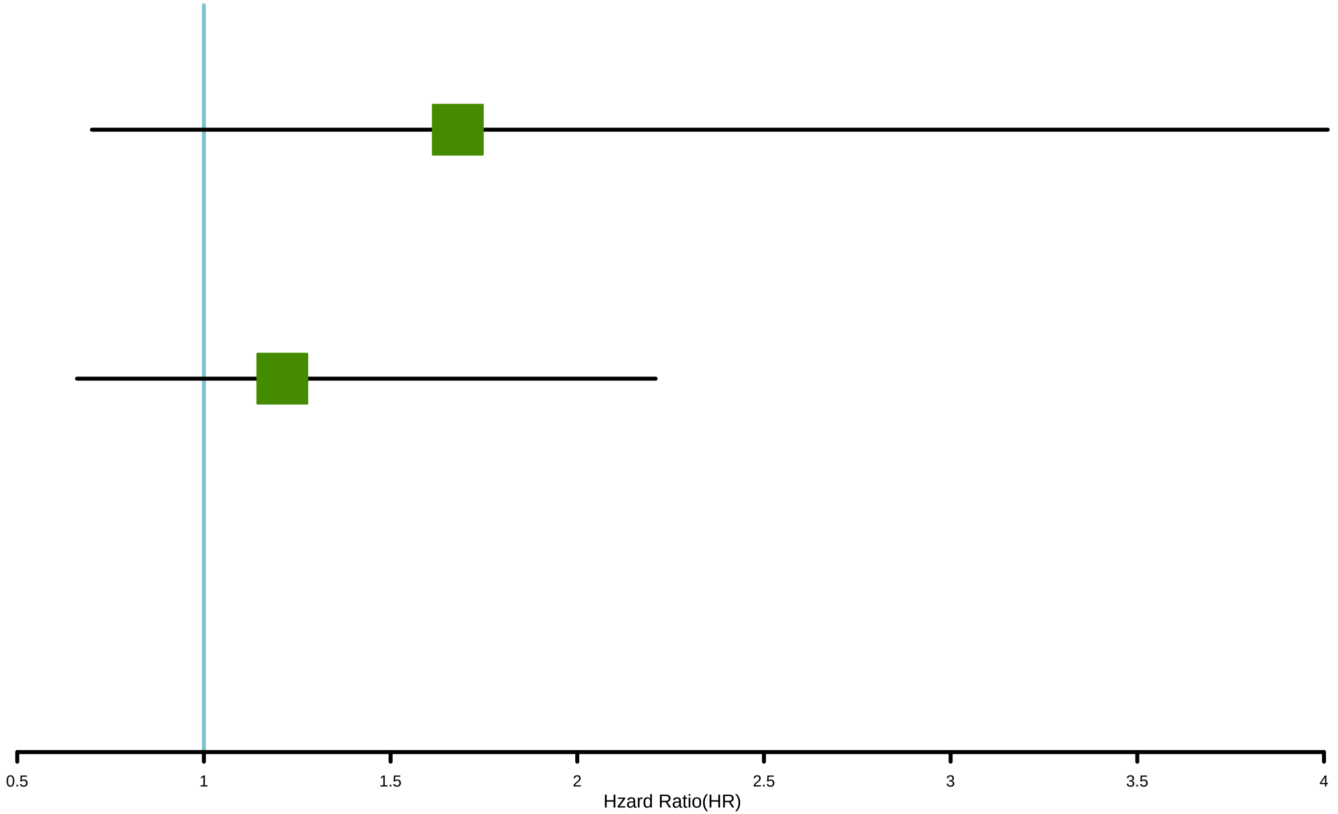

I

Subgroup	No. of patients	P value	HR(95%CI)
TERT	77	0.001	0.11(0.03−0.39)
CDKN2A/B	77	0.003	3.74(1.54−9.07)

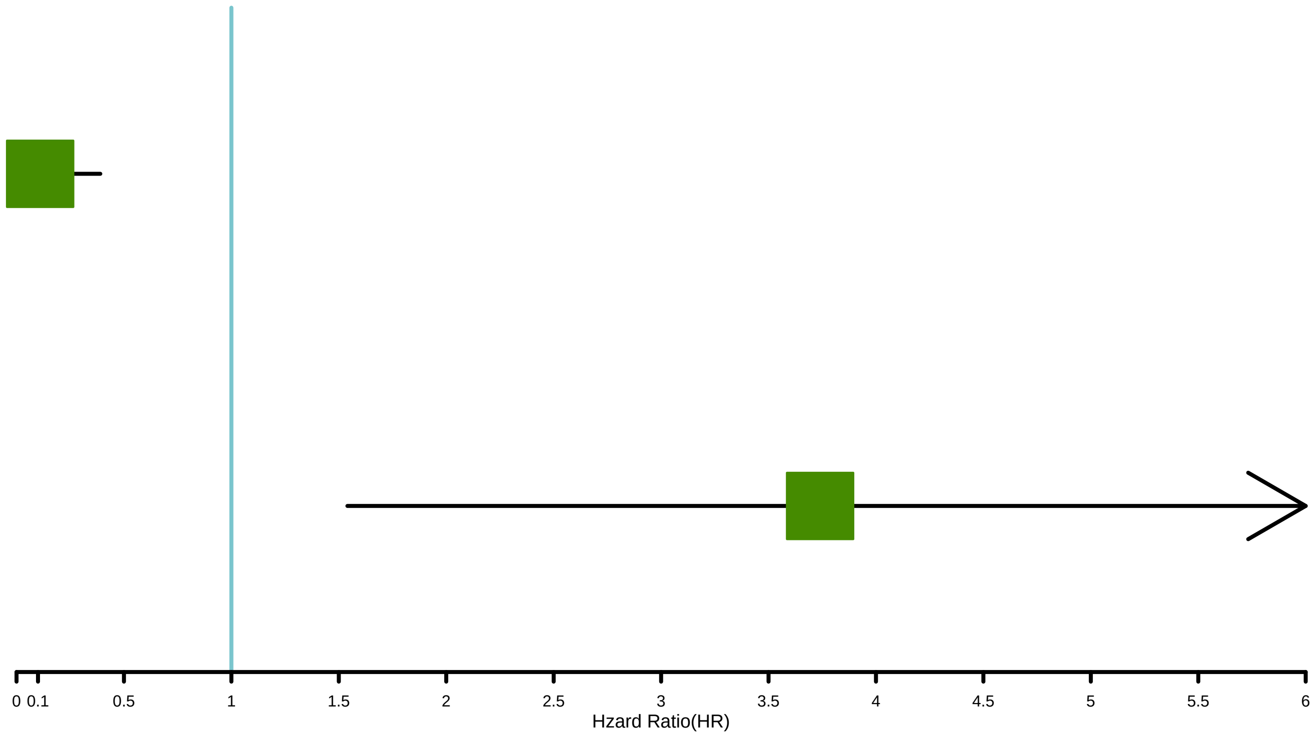

m

Subgroup	No. of patients	P value	HR(95%CI)
TERT	69	0.245	1.68(0.7−4.01)
CDKN2A/B	69	0.657	1.16(0.61−2.2)

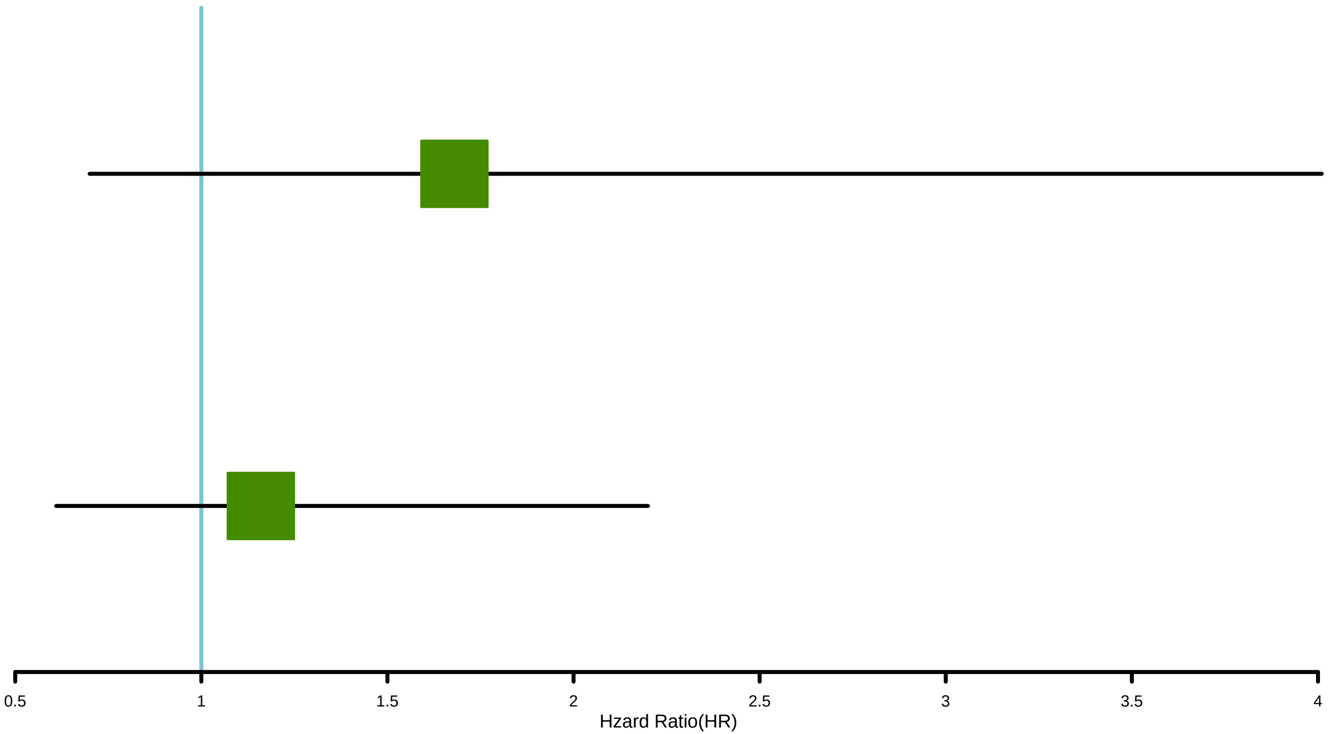

Supplement: Supplementary file 1 — Data S1: [file CAM4-14-e70533-s001.zip › cam470533-sup-0006-FigureS6.pdf]
