# Supplementary material for: TERT p Mutation and its Prognostic Value in Glioma Patients Under the 2021 WHO Classification: A Real‐World Study
Source: Cancer Med. 2025 Jan 13;14(2):e70533. doi: 10.1002/cam4.70533 (PMC11727134; doi:10.1002/cam4.70533)
Supplement: Supplementary file 1 — Data S1: [file CAM4-14-e70533-s001.zip › cam470533-sup-0002-FigureS2.docx]

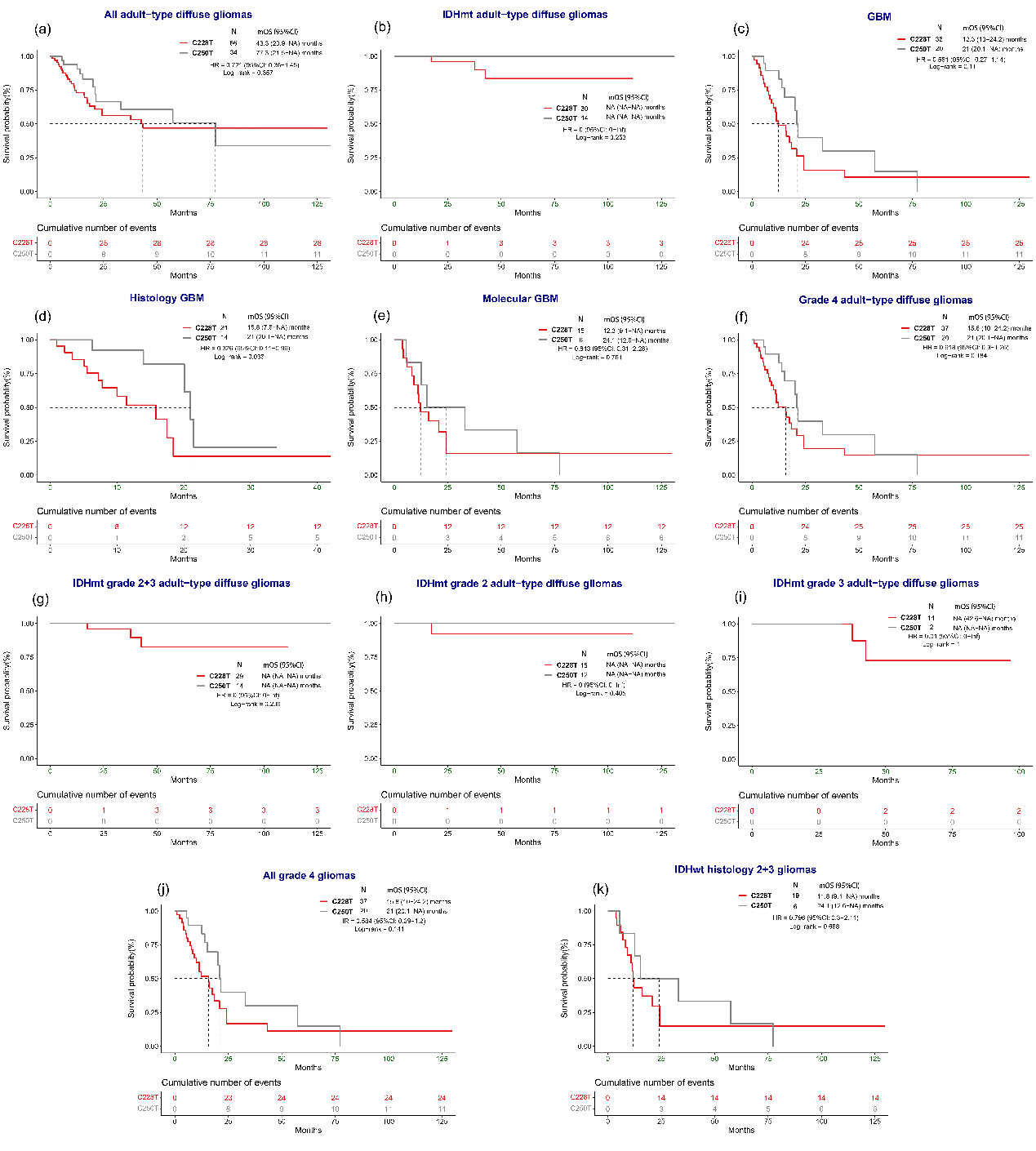
**Supplementary Figure 2. Overall survival of TERT promoter C228T and C250T mutation in different subtypes gliomas.**

(a) In all adult-type gliomas. (b) In IDH mutant adult-type diffuse gliomas. (c) In glioblastomas. (d-e) In histology GBM and molecular GBM. (f) In all grade 4 adult-type gliomas including both IDH wildtype and mutant. (g) In IDH mutant grade 2 and 3 glioma. (h-i) In IDH mutant grade 2 or grade 3 adult-type gliomas, respectively. (j) In all grade 4 gliomas, including pediatric type. (k) In IDH wildtype glioma with histological grade 2 and 3 appearance. GBM, glioblastoma. mOS, median OS. IDHmt, IDH mutant .IDHwt, IDH wildtype.
