# Supplementary material for: TERT p Mutation and its Prognostic Value in Glioma Patients Under the 2021 WHO Classification: A Real‐World Study
Source: Cancer Med. 2025 Jan 13;14(2):e70533. doi: 10.1002/cam4.70533 (PMC11727134; doi:10.1002/cam4.70533)
Supplement: Supplementary file 1 — Data S1: [file CAM4-14-e70533-s001.zip › cam470533-sup-0003-FigureS3.docx]

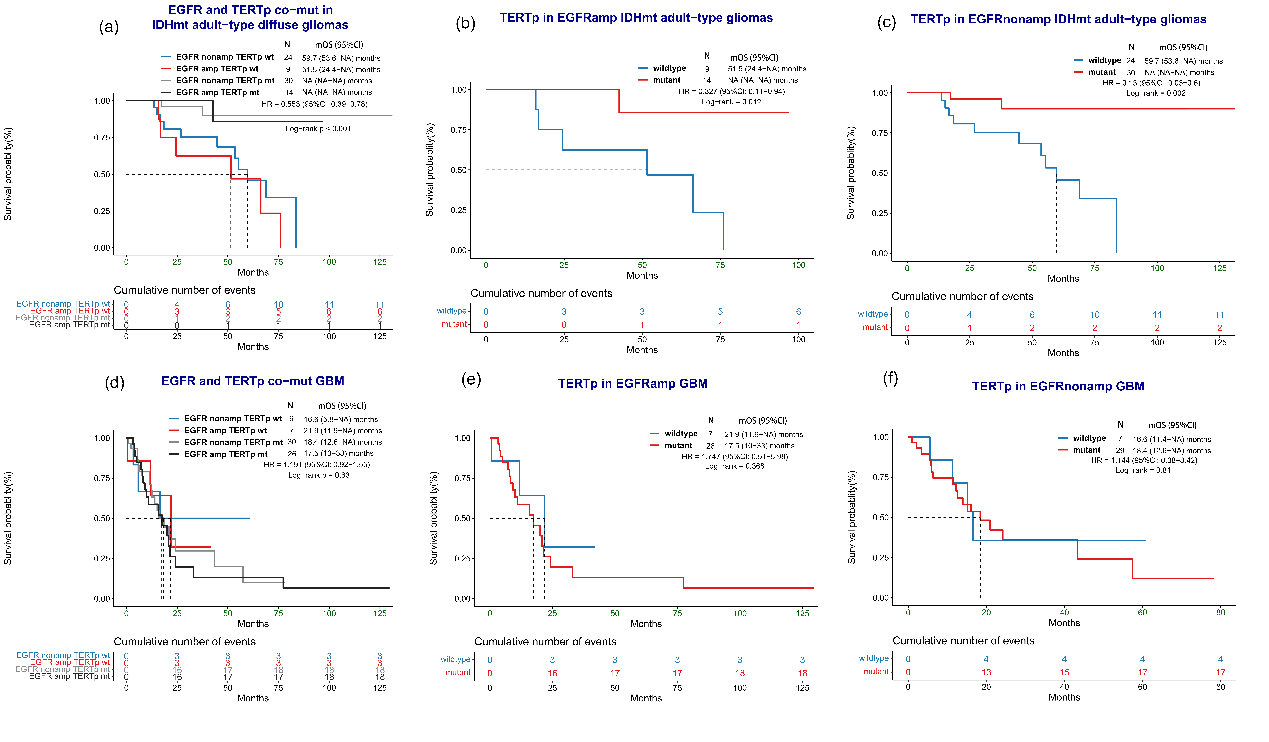
**Supplementary Figure 3. Overall survival of TERT promoter mutation in different EGFR and IDH status adult-type glioma.**

(a) In all IDH mutant adult-type gliomas with different EGFR status. (b) In EGFR amplification adult-type gliomas with IDH mutant. (c) In EGFR non-amplification adult-type gliomas with IDH mutant. (d) In all GBM with different EGFR status. (e) In EGFR amplification GBM. (f) In EGFR non-amplification GBM.
