# Supplementary material for: TERT p Mutation and its Prognostic Value in Glioma Patients Under the 2021 WHO Classification: A Real‐World Study
Source: Cancer Med. 2025 Jan 13;14(2):e70533. doi: 10.1002/cam4.70533 (PMC11727134; doi:10.1002/cam4.70533)
Supplement: Supplementary file 1 — Data S1: [file CAM4-14-e70533-s001.zip › cam470533-sup-0004-FigureS4.docx]

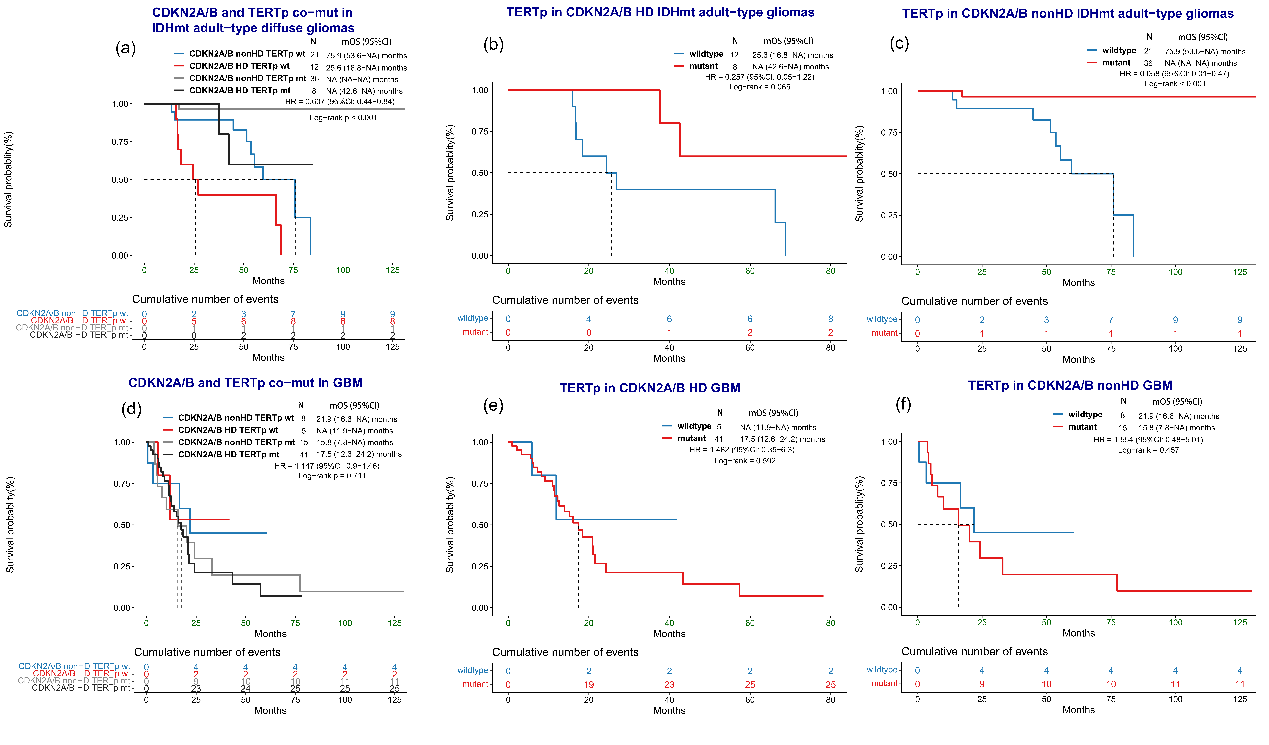


**Supplementary Figure 4. Overall survival of TERT promoter mutation in different CDKN2A/B and IDH status adult-type glioma.**

(a) In all IDH mutant adult-type gliomas with different CDKN2A/B status. (b) In CDKN2A/B homozygous deletion adult-type gliomas with IDH mutant. (c) In CDKN2A/B non-homozygous deletion adult-type gliomas with IDH mutant. (d) In all GBM with different CDKN2A/B status. (e) In CDKN2A/B homozygous deletion GBM. (f) In CDKN2A/B non-homozygous GBM.
