# Supplementary figures and images for: TERT p Mutation and its Prognostic Value in Glioma Patients Under the 2021 WHO Classification: A Real‐World Study
Source: Cancer Med. 2025 Jan 13;14(2):e70533. doi: 10.1002/cam4.70533 (PMC11727134; doi:10.1002/cam4.70533)

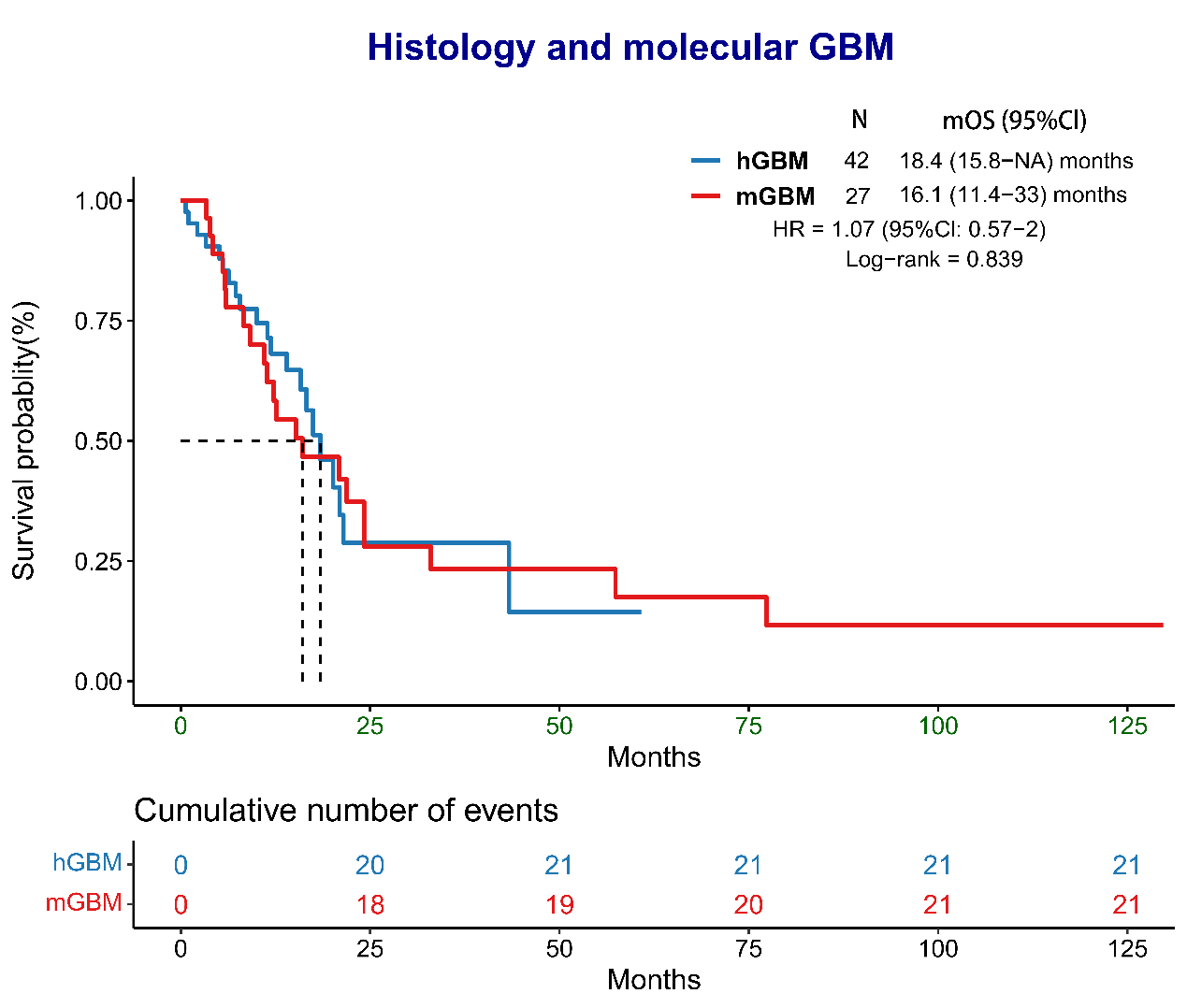


**Supplementary Figure 1. Overall survival of histology GBM and molecular GBM.**

GBM, glioblastoma.

Supplement: Supplementary file 1 — Data S1: [file CAM4-14-e70533-s001.zip › cam470533-sup-0001-FigureS1.docx]
